# Marine diesel engine reliable intelligent fault diagnosis method based on the generalized multi-source information fusion

**DOI:** 10.1016/j.isci.2025.114345

**Published:** 2025-12-05

**Authors:** Zaimi Xie, Chunmei Mo, Baozhu Jia

**Affiliations:** 1 Naval Architecture and Shipping College, Guangdong Ocean University, Zhanjiang, China; 2 Technical Research Center for Ship Intelligence and Safety Engineering of Guangdong Province, Zhanjiang, China; 3 Guangdong Provincial Key Laboratory of Intelligent Equipment for South China Sea Marine Ranching, Zhanjiang, China; 4 College of Electronic and Information Engineering, Guangdong Ocean University, Zhanjiang, China

**Keywords:** simulation of computer system, engineering, energy modelling, systems engineering

## Abstract

Diesel engines provide essential power and energy guarantee for vessels. Due to scarce fault samples and complex parameter-fault coupling, traditional methods struggle in marine diesel engine diagnosis, underscoring the need for reliable intelligent approaches based on multi-source thermal parameter fusion. This article develops a reliable intelligent fault diagnosis method based on generalized multi-source information fusion. Key parameters are selected using Pearson correlation and mutual information, while an improved Bayesian optimization algorithm automatically tunes random forest parameters to enhance accuracy. TreeSHAP interprets parameter influence, guiding feature selection for retraining. An improved Dempster-Shafer evidence fusion strategy with Shannon entropy and Jousselme distance strengthens model decision-making. The method achieves 99.45% accuracy, outperforming existing models in F1-score and recall, and identifies critical thermal parameters such as intercooler velocity, maximum pressure during combustion, brake power, and velocity of the exhaust manifold. This approach provides a reliable, interpretable, and robust diagnostic tool for marine diesel engines.

## Highlights

• Generalized multi-source fusion framework for marine engine diagnosis is developed
• TreeSHAP interprets model decisions and guides feature selection
• PCEI-based Bayesian optimization tunes model hyperparameters automatically
• Enhanced Dempster-Shafer evidence fusion ensures robust, accurate model decisions

## Graphical abstract

**Figure:**
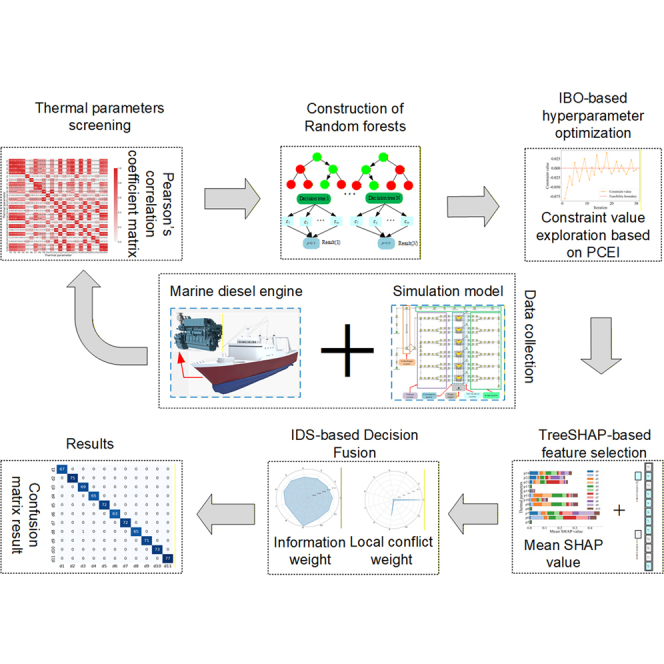

## Introduction

Diesel engines constitute the principal propulsion system for vessels and provide essential power for diverse onboard equipment. However, their operational stability can be significantly affected by variations in sea state and internal system dynamics, thereby introducing multiple potential failure modes.^1^ These faults often cause abnormal fluctuations in diesel engine thermal parameters, pushing them beyond the allowable range and resulting in performance degradation, functional degradation of each subsystem, and even serious faults.^2^ However, due to the scarcity of real ship fault samples, it is challenging to rely solely on measured data for comprehensive condition monitoring and diagnosis. Therefore, it is necessary to build a dynamic simulation model based on a real ship diesel engine and extract thermal parameters from each subsystem to assist in fault diagnosis and condition assessment. However, there is usually no one-to-one correspondence between thermal parameters and faults, and the multi-subsystem interaction makes the relationship complex and unclear. Based on this, this study proposes an interpretable intelligent fault diagnosis method for thermal parameters to reveal the association rules between various thermal parameters and realize the accurate identification of fault states under the interaction of multiple subsystems.

Currently, pertinent research emphasizes the application of machine learning in diesel engine fault detection, with the Support Vector Machine (SVM)^3^ facilitating the diagnosis of faults in diesel engine combustion systems, thereby achieving enhanced diagnostic accuracy. Similarly, it is also applied to solve air conditioning system fault diagnosis problems.^4^ To address the inadequacy of SVM in processing high-dimensional, complex data, the Gaussian mixture model^5^ may efficiently identify faults in diesel engine systems. The input data that follows a Gaussian distribution. The artificial neural network^6^ utilizes thermal factors that deviate from the Gaussian distribution as the foundation for diagnosing diesel engine issues. These approaches exhibit low root-mean-square error and standard error, effectively managing complex mapping; however, they are prone to overfitting in small sample sizes. Conventional models, such as K-nearest neighbors (KNNs), frequently produce uncalibrated output probabilities and are vulnerable to noise and extraneous information. Random Forest (RF)^7^ demonstrates superior classification efficacy for diagnosing faults in satellite attitude control systems, making it well-suited for small-sample fault diagnostics and exhibiting enhanced robustness against noise and outliers. In recent years, deep learning (DL) has been widely adopted in intelligent fault diagnosis due to its strong ability to automatically learn complex nonlinear features, particularly excelling in multi-sensor information fusion and adaptation to complex operating conditions. For example, MACCDAN,^8^ MRCFN,^9^ CDTFAFN,^10^ and MIFDELN.^11^ However, deep models typically require large-scale labeled data, involve complex training, and offer limited interpretability. By contrast, traditional machine learning methods remain robust and interpretable in small-sample or domain-specific scenarios, allowing clear identification of key parameters influencing faults. Therefore, for diesel engine fault diagnosis tasks with limited data and a need for interpretability, random forest (RF) represents a more suitable choice.

However, in traditional models such as random forests, hyperparameters are usually set manually, which limits both computational efficiency and diagnostic accuracy. The Bayesian optimization algorithm^12^ can adaptively refine hyperparameters to achieve optimal values; however, the traditional Expectation Improvement Criterion (EI) is prone to falling into the unrealistic exploration zone in multi-objective optimization. The aforementioned research findings significantly advance the development and enhancement of ship fault diagnosis theory. However, Random Forest randomly selects a subset of features when constructing decision trees. This approach makes it difficult to interpret the influence of individual features on the target class, as the feature selection process exhibits certain black box characteristics. Furthermore, the uncertainty and potential conflicts among evidence sources are often overlooked during the information fusion process, which may compromise the reliability of the model’s evaluation outcomes. Therefore, the Tree SHapley Additive exPlanations (Tree SHAP)^13^ can be used to calculate the contribution of each parameter to the target category and visualize the importance of the parameter. This method is used in many fields.^14^

After optimizing the model’s feature selection through the TreeSHAP method, further improvement can be achieved at the decision level by enhancing the fusion mechanism. Some studies introduce Dempster-Shafer (D-S) evidence theory to alleviate the above problems, so as to improve the accuracy and reliability of fault diagnosis. The Dempster-Shafer (D-S) theory^15^ effectively elucidates the multi-source information fusion process and addresses uncertainty in contrast to deep learning methodologies. However, the traditional Dempster combination rule often yields counterintuitive results when handling highly conflicting evidence, thereby weakening the accuracy of evidence combination. Ref.^16^ employs a weighing approach to assess the conflict among bodies of evidence, neglecting the volume of information inside those bodies. Ref.^17^ presents Belief Sinkhorn Distance (BSD) in conjunction with Dunn’s entropy, while ref.^18^ discusses Jensen-Shannon^19^^,^^20^ dispersion integrated with belief entropy. These methods effectively improve the accuracy of fusion results by dealing with the conflict between evidence. Although improved methods—such as conflict-aware weighting, entropy-based measures, and divergence metrics—address some limitations, they often overlook the structural complexity of internal evidence, lack sensitivity to non-specific focal elements, or suffer from high computational complexity. This highlights the need for a more efficient, interpretable, and evidence-structure-aware belief fusion framework.

Although considerable research has been conducted, and some results have been achieved in intelligent fault diagnosis of diesel engines. There are still some challenges that need to be addressed: (1) Manually setting hyperparameters may significantly affect the fault classification accuracy of the model. (2) Random forest has randomness in parameter selection, does not consider the contribution of parameters to the target category, and lacks visual interpretation. (3) It ignores the structural complexity of internal evidence, lacks sensitivity to non-focus elements, and does not fully calculate the amount of global and local information and conflict.

This article mostly encapsulates the subsequent aspects: (1) This article presents a method based on IBO-RF that utilizes Penalization Constrained Optimization EI (PCEI), an improved Bayesian Algorithm (IBO), which executes hyper-parameter optimization, including the number of decision trees and the maximum depth in the RF. (2) The SHAP method was used to quantify the contribution of each parameter to the target category, and it was used as the criterion of random forest parameter selection. (3) Based on the information quantity sensitivity and similarity drive, a new evidence weight factor is designed to improve the accuracy of evidence fusion. (4) This research presents an innovative, intelligent fault diagnosis framework for marine diesel engines that utilizes IBO-RF-IDS multi-source information fusion. It can enhance the accuracy of fault diagnostics.

## Results and discussion

### Experimental verification

**Figure 1:**
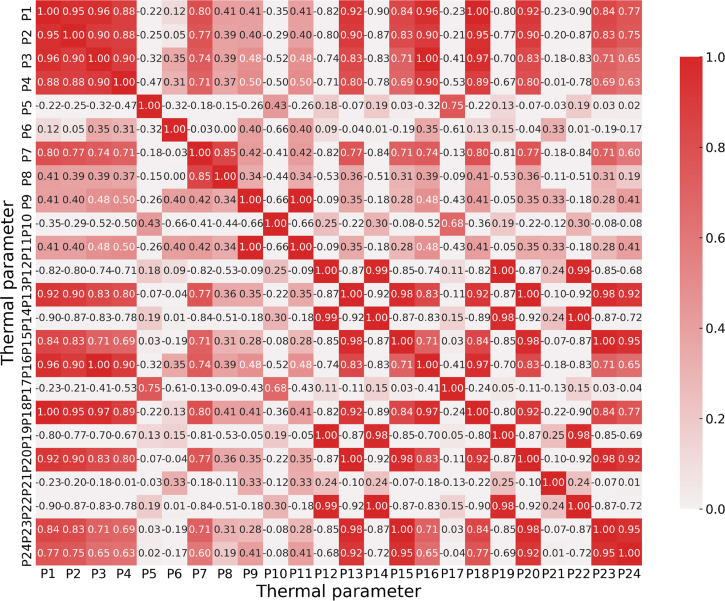
Pearson’s correlation coefficient matrix for thermal parameters

**Figure 2:**
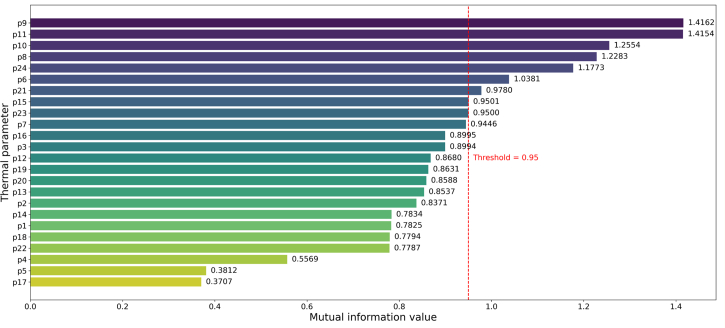
Mutual information values between parameters and target categories

The sample set of 24 parameters is collected from different subsystems in a marine diesel engine, and there are interactions between the systems. The correlation matrix between the thermal parameters was obtained in this study by the Pearson correlation coefficient method, and the results are shown in Figure 1. These methods can increase the explainability of each system parameter. When the correlation coefficient exceeds 0.9, it indicates a strong correlation between the parameters. Specifically, parameters p1, p2, p3, p4, p9, p12, p13, p14, p15, p16, p18, p19, p20, and p23 exhibit strong correlation. Conversely, it is considered that there is low correlation, and the parameters with low correlation include p5, p6, p7, p8, p10, p11, p17, and p21. If all parameters are used as model inputs, it will significantly increase the model computation. If only strong or weak correlation parameters are selected, it may lead to a loss of some critical fault information and an increase in redundant information. The mutual information values between parameters and target categories are illustrated in Figure 2. For this reason, the contribution of each parameter to the target category is measured by mutual information, and the threshold is set to 0.95 to filter the high contribution parameters, including p6, p8, p9, p10, p11, p15, p21, and p24. This article considers avoiding the loss of important parameter information. The low correlation parameters p5, p7, p17, and p22 were taken into account. Twelve parameters, such as p6, were selected as inputs to the model in this study. The analysis of the experimental results showed that most of the parameters showed low correlation, but there was a significant and strong correlation between p9 and p15 (which may lead to information redundancy). Overall, the Pearson correlation coefficient and mutual information can reduce the parameter dimension. Based on the differences in the contribution of these parameters to the classification model, the following two aspects need to be further addressed: 1) the uncertainty performance of the parameters in different conditions; and 2) the specific influence mechanism of the parameters on the accuracy of fault diagnosis. Consequently, this study proposes an IDS-based multi-source information fusion method to enhance the fault diagnosis accuracy of diesel engines.

### Multi-source information fusion method

**Figure 3:**
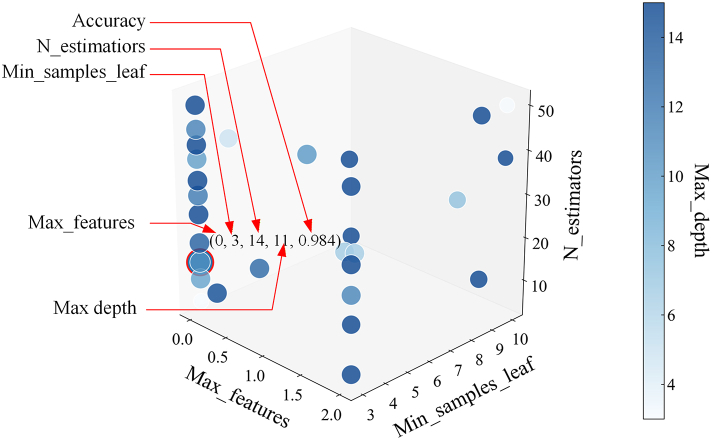
IBO-based optimization of RF hyperparameters

The Bayesian algorithm is initialized with 30 iterations and 5-fold cross-validation, using the accuracy rate as a metric to optimize hyperparameters. The accuracy rate serves as a metric to optimize the hyperparameters. The search hyperparameters are the number of decision trees, maximum depth, control center point coefficient, and control slope coefficient. The number of decision trees (n_estimators) ranges from 5 to 50, the maximum tree depth (max_depth) from 3 to 15, and the maximum number of features (max_features) is coded as 0 for ‘sqrt’, 1 for ‘log2’, and 2 for 0.5. The minimum number of samples per leaf (min_samples_leaf) ranges from 3 to 10. The IBO-based optimization of the RF model over 30 iterations is shown in Figure 3. The accuracy of the IBO-RF-IDS method reaches its highest value when the four hyperparameters of the number of max_features, min_samples_leaf, n_estimators, and max_depth are 0, 3, 14, and 11, respectively, and the accuracy is 0.984.

**Figure 4:**
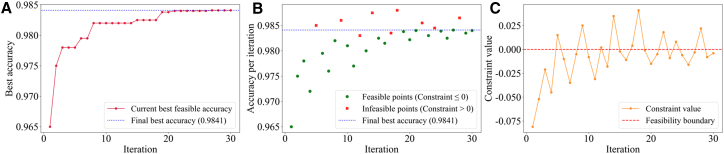
The process of IBO optimizing RF hyperparameters (A) Best feasible accuracy over iterations. (B) Overview of sampled points. (C) Constraint value exploration.

The IBO optimization process of RF hyperparameters and its effectiveness are illustrated in Figure 4. As shown in Figure 4A, the algorithm rapidly improves performance within the first ten iterations, then stabilizes and converges to an optimal accuracy of 0.9841, indicating efficient global search capability. As shown in Figures 4B and 4C, the method exhibits an intelligent convergence strategy. A clear trade-off between performance and constraints (Figure 4B), accompanied by convergence of the most constrained values converge near zero, indicating accurate learning of the feasible boundary (Figure 4C). Overall, the algorithm efficiently explores constraint boundaries, avoids infeasible regions, and achieves the optimal feasible solution.

**Figure 5:**
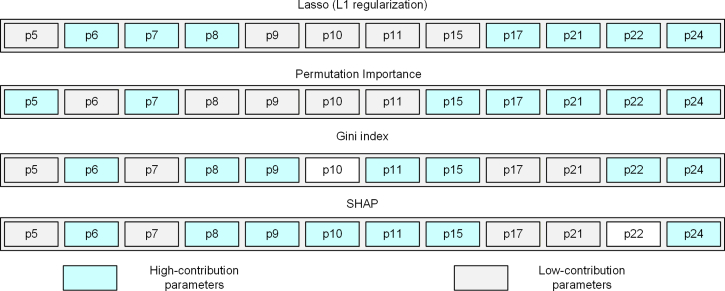
Optimal subset of different feature selection methods in decision tree 1

To enable a comprehensive comparative analysis, multiple feature selection methods were employed, and the optimal parameter subsets identified by each method are visually presented in Figure 5. Using IBO-RF-IDS as the classification model, the accuracy achieved by the Lasso (L1 regularization), Gini Index, Permutation Importance, and SHAP methods was 83.42%, 85.62%, 89.22%, and 94.80%, respectively. These results demonstrate that the parameter subset selected via the SHAP method offers superior diagnostic performance compared to those obtained through the other feature selection approaches. In summary, the SHAP method has advantages in RF feature selection.

**Figure 6:**
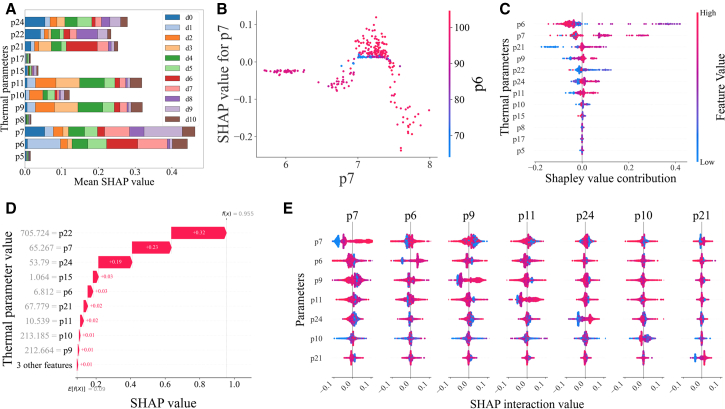
Fault analysis of the intake valve leakage (d8) based on SHAP values (A) Global parameter importance based on mean SHAP values across output classes. (B) Dependence plot shows the marginal effect of p7 and its interaction with p6. (C) Beeswarm plot displays the distribution and directional impact of top features. (D) Waterfall plot illustrates local feature contributions for a single prediction instance. (E) Matrix of SHAP interaction values among key thermal parameters.

The fault analysis of the intake valve leakage (d8) based on SHAP values (Figure 6). The SHAP values of the 12 parameters across eleven conditions are presented in Figure 6A. Features p6, p7, p9, and p11 are identified as the key factors influencing the model’s predictions. Taking state d8 (intake valve leakage) as an example, features p6, p7, p9, and p21 exhibit particularly strong contributions. The nonlinear interaction between p6 and p7 shows that higher values of p7 have a positive effect on prediction only when p6 remains at a low level (Figure 6B). As shown in Figure 6C, the overall impact of all features, where p6 and p7 emerge as the most influential, followed by p21 and p22, is consistent with the findings in Figure 6A. As shown in Figure 6D, the contribution of each feature for a specific sample is presented, where E(f(x)) denotes the model’s mean prediction (0.09), and f(x) represents the predicted value for this sample (0.955). All features contribute positively in this case, with p22 providing the largest effect. The interaction effects among the seven most important features, where diagonal entries represent main effects and off-diagonal entries capture pairwise interactions (Figure 6E). The results reveal a pronounced interdependence between p6 and p7 under the d8 condition. The model not only relies on the strong main effects of p6 and p7 but also dynamically modulates the contributions of p9 and p21 through p10, thereby enhancing the accuracy of fault prediction.

The entropy weights of BPA for the 11 categories obtained by 14 decision trees in the test samples are shown in Table 1. As can be seen from the table, most decision trees assign a high prediction probability (close to 1) to state d1, and the entropy weight is less than 0.2, indicating that these decision trees have a very high recognition degree for d1. However, some decision trees (such as the 5th, 6th, 10th, and 11th) predict d1 with a probability of about 0.5, and their entropy weights are all greater than 1, indicating that these decision trees have high uncertainty in the decision results. These observation results can provide a reference for improving the rules of evidence combination.

**Table 1:** BPA and entropy of decision trees under different conditions in the test sample

Trees	d1	d2	d3	d4	d5	d6	d7	d8	d9	d10	d11	Entropy
DT1	1	0	0	0	0	0	0	0	0	0	0	0
DT2	1	0	0	0	0	0	0	0	0	0	0	0
DT3	1	0	0	0	0	0	0	0	0	0	0	0
DT4	0.964	0	0	0	0	0	0	0.036	0	0	0	0.222
DT5	0.529	0.059	0	0	0	0	0	0	0.353	0	0.059	1.497
DT6	0.417	0	0.083	0	0.017	0	0.133	0.2	0.083	0.033	0.033	2.401
DT7	0.974	0	0	0	0	0	0	0	0.026	0	0	0.172
DT8	1	0	0	0	0	0	0	0	0	0	0	0
DT9	1	0	0	0	0	0	0	0	0	0	0	0
DT10	0.6	0	0	0.04	0	0	0	0	0.36	0	0	1.1585
DT11	0.575	0	0	0.05	0	0	0.025	0	0.35	0	0	1.338
DT12	1	0	0	0	0	0	0	0	0	0	0	0
DT13	1	0	0	0	0	0	0	0	0	0	0	0
DT14	1	0	0	0	0	0	0	0	0	0	0	0

The BPA fusion results for the 11 categories from 14 decision trees in a sample using the traditional D-S evidence theory combination rule are shown in Table 2. As can be seen from the table, the fusion results of the first four decision trees exhibit high confidence for state d1. However, when the 5th, 6th, and 11th decision trees are added, the fusion results fluctuate, leading to a paradox. Specifically, the addition of the 11th decision tree causes the fusion results to fluctuate, resulting in a paradoxical outcome when the 5th decision tree is fused. This shows that the traditional D-S evidence theory is easily affected by the conflict evidence, and may produce the wrong conclusion of overconfidence in the face of conflict information, ignoring the information contained in the conflict itself, making the fusion process fragile and the resulting judgment too decisive, which may lead to a wrong decision.

**Table 2:** BPA after decision trees fusion in the t1 sample

	d1	d2	d3	d4	d5	d6	d7	d8	d9	d10	d11
f1	1	0	0	0	0	0	0	0	0	0	0
f2	1	0	0	0	0	0	0	0	0	0	0
f3	1	0	0	0	0	0	0	0	0	0	0
f4	0.001	0	0	0	0	0	0	0	0.999	0	0
f5	0.993	0.001	0.001	0.001	0	0	0.001	0.002	0	0	0.001
f6	1	0	0	0	0	0	0	0	0	0	0
f7	1	0	0	0	0	0	0	0	0	0	0
f8	1	0	0	0	0	0	0	0	0	0	0
f9	1	0	0	0	0	0	0	0	0	0	0
f10	0.994	0	0	0	0	0	0	0	0.006	0	0
f11	1	0	0	0	0	0	0	0	0	0	0
f12	1	0	0	0	0	0	0	0	0	0	0
f13	1	0	0	0	0	0	0	0	0	0	0

Note: “f1-f13” represents the results obtained by IDS after pairwise fusion of each decision tree.

**Figure 7:**
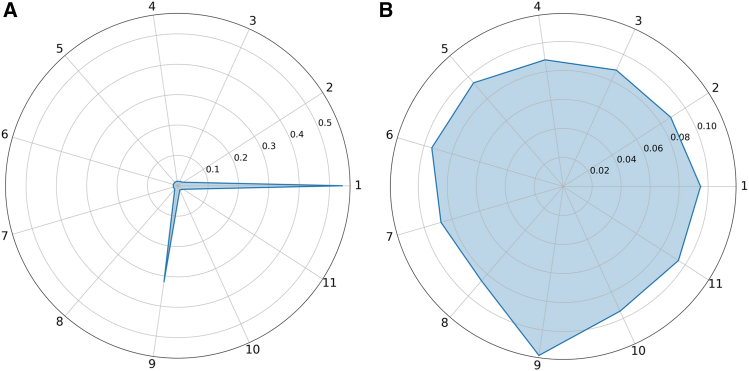
Information weight coefficient and local conflict weight coefficient (A) Information weight coefficients across the indices. (B) Local conflict weight coefficients across the indices.

The weight coefficient of information for a single decision tree across 11 states, as well as its local conflict weight coefficient in relation to other decision trees, based on a specific sample (Figure 7). As shown in Figure 7A, the information weights of states d1 and d9 are significantly higher, indicating that this decision tree carries the largest amount of information for these two states during the fusion process. As illustrated in Figure 7B, these two states still have high local conflict weights across decision trees. By weighting the information weight and the local conflict weight, the conflict discount coefficient between decision trees can be obtained. One part of the conflict is used to improve the fusion accuracy, and the other part is used as a measure of the reliability of the decision result.

The fusion results of decision trees for 11 categories in a sample based on the evidence combination rule improved by weighting information weight and local conflict weight, are shown in Table 3. As can be seen from the table, this method does not produce paradoxes in the fusion process, and state d1 always maintains the highest predicted probability, while the overall conflict is gradually reduced. Even if the individual decision trees have high conflict, the final fusion result still effectively improves the prediction probability. This demonstrates that the information weighting coefficient and the local conflict weighting coefficient effectively mitigate the uncertainty resulting from evidence conflict, thereby enhancing the robustness of the decision process. Although the belief value fluctuates in the fusion process, the overall trend converges to the correct category smoothly and monotonically, showing good convergence characteristics.

**Table 3:** BPA and conflict after decision trees fusion in the test sample

	d1	d2	d3	d4	d5	d6	d7	d8	d9	d10	d11	Conflict
f1	1	0	0	0	0	0	0	0	0	0	0	0
f2	1	0	0	0	0	0	0	0	0	0	0	0
f3	0.98	0	0	0	0	0	0	0	0	0.001	0	0.018
f4	0.741	0.001	0.001	0.001	0	0	0.002	0.002	0.015	0	0.001	0.236
f5	0.672	0.001	0.001	0.001	0	0	0.002	0.004	0.02	0	0.001	0.297
f6	0.962	0	0	0	0	0	0	0	0.003	0	0	0.034
f7	0.998	0	0	0	0	0	0	0	0	0	0	0.002
f8	1	0	0	0	0	0	0	0	0	0	0	0
f9	0.781	0.001	0.001	0.001	0	0	0.001	0.002	0.0012	0	0.001	0.2
f10	0.757	0.001	0.001	0.001	0	0	0.001	0.002	0.019	0	0.001	0.217
f11	0.982	0	0	0	0	0	0	0	0.001	0	0	0.017
f12	0.999	0	0	0	0	0	0	0	0	0	0	0.001
f13	1	0	0	0	0	0	0	0	0	0	0	0

The BPA and conflict values of each category obtained by the fusion of all decision trees in different samples are shown in Table 4. As can be seen from the table, the conflict values of most samples are lower than 0.1, and the predicted probability of the actual state after fusion is close to 1, showing an obvious dominant decision. The conflict value of some samples (such as t5 and t9) is at a medium level, but the confidence is still more than 0.65, indicating that the model can still stably reflect the decision-making tendency in the presence of uncertainty, avoid the wrong judgment of overconfidence, and reflect the robustness of the method in a fuzzy environment.

**Table 4:** BPA and conflict after decision tree fusion in some samples

	d1	d2	d3	d4	d5	d6	d7	d8	d9	d10	d11	Conflict
t1	1	0	0	0	0	0	0	0	0	0	0	0
t2	0	0	0	0	0	0	1	0	0	0	0	0
t3	0	0	0	0	1	0	0	0	0	0	0	0
t4	0	0	0	0	0	0	1	0	0	0	0	0
t5	0.007	0.008	0.002	0.006	0.001	0	0.007	0.002	0	0	0.775	0.194
t6	0	0	0	0	0	0	0	0	0	0	1	0
t7	0	0	0	0	0	0	0	0	1	0	0	0
t8	0	0	0	0	0	1	0	0	0	0	0	0
t9	0	0.006	0.002	0.001	0.001	0	0.002	0	0	0	0.669	0.318
t10	0	0	0	0	0	0	0	0	0	1	0	0

The two metrics of information fusion, namely entropy and efficiency index, are utilized to evaluate the effectiveness of the IBO-RF-IDS method in multi-source information fusion. The information fusion entropy and efficiency index of some decision trees and fusion results are shown in Table 5. The fusion result 1 shows improvements of 27.8% and 28.8% in the information fusion entropy metric, and 32.8% and 47% in the efficiency index, compared to tree 1 and tree 2, respectively. The fusion result 2 improved 27% and 51.8% in the metric of information fusion entropy, as well as 13.9% and 83.1% in the effective index compared with the fusion results 1 and tree 3, respectively. The fusion result 3 showed improvements of 20.5% and 64.5% in the information fusion entropy metric, as well as 5.4% and 115.7% in the effective index, compared to fusion results 2 and tree 4, respectively. With a lower metric of information fusion entropy, the effective index is higher, indicating that the IBO-RF-IDS method is more effective for multi-source information fusion results.

**Table 5:** Information fusion entropy and effective index

	Decision trees				Fusion result		
	1	2	3	4	F1	F2	F3
Information fusion entropy	1.837	1.862	2.009	2.168	1.326	0.969	0.770
Efficiency index	0.516	0.466	0.426	0.381	0.685	0.780	0.822

**Figure 8:**
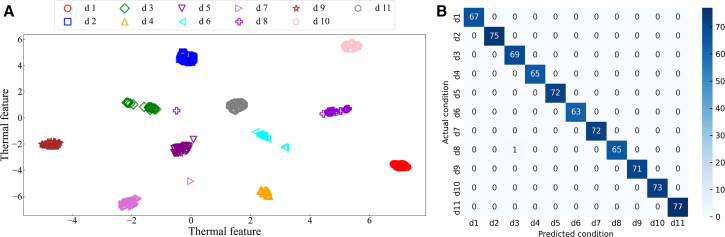
Performance evaluation of the IBO-RF-IDS method for fault diagnosis (A) t-SNE visualization illustrates the sample distribution and clustering of different fault conditions. (B) Confusion matrix displays the classification accuracy and prediction results.

In order to validate the effectiveness of the BO-RF-IDS method for diesel engine fault diagnosis. The evaluation results of the IBO-RF-IDS method in fault diagnosis (Figure 8). The partial sample distribution diagram after *t-*SNE dimensionality reduction is shown in Figure 8A. The parameter sample points of each condition are well aggregated. There are a few conditions where sample points are misdiagnosed as other conditions. For example, d7 and d4, d8 and d3. However, the different condition classifications are obvious. In this method, the number of misdiagnosis errors in condition d7 and condition d8 is 1. As shown in Figure 8B, most of the sample points in the state are on the diagonal of the confusion matrix. The results demonstrate the effectiveness of the IBO-RF-IDS multi-source information fusion method for fault diagnosis. It can improve the performance of fault diagnosis.

**Figure 9:**
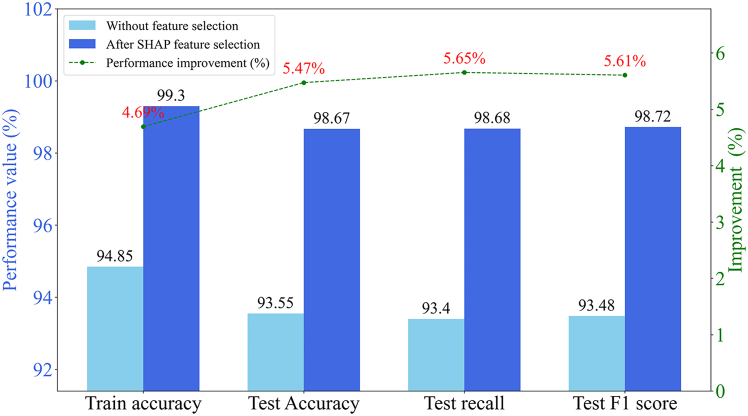
Comparison of model performance before and after SHAP-based feature selection The bars indicate the absolute metric values, while the green line (right Y axis) represents the percentage improvement in model performance.

As depicted in Figure 9, the experimental results demonstrate that SHAP-based feature selection substantially improves model performance. Following feature optimization, the model’s test accuracy, recall, and F1 score increased from approximately 93.5%–98.67%, 98.68%, and 98.72%, respectively, with an average improvement exceeding 5.5%, highlighting strong generalization to unseen data. Training accuracy also rose to 99.3% and closely matches the test performance, indicating high-quality fitting rather than overfitting after removing redundant features. Overall, SHAP feature selection effectively streamlines the feature space, allowing the model to focus on core information and thereby significantly enhancing prediction accuracy, generalization, and robustness, confirming its effectiveness as an efficient model optimization strategy.

Taken together, the IBO approach enhances the computing efficiency of the model. In this article, a joint weight mechanism of information quantity and conflict based on Shannon entropy and JS distance is proposed to effectively suppress the interference of unreliable decisions on the final result, and achieve better diagnosis performance in complex multi-fault scenarios. Additionally, in the specific fault case (d8), the contributions and interactions of thermodynamic parameters are systematically investigated, accompanied by interpretive analyses that elucidate the model’s decision-making outcomes. According to findings from the IBO-RF-IDS model estimation and SHAP analysis. The intercooler outlet velocity, maximum combustion burst pressure, brake power, and exhaust manifold outlet velocity are the key thermodynamic parameters affecting the fault diagnosis results. The observed interdependence between intercooler outlet velocity and maximum combustion burst pressure, together with the dynamic modulation of brake power and exhaust manifold outlet velocity through outlet temperature, demonstrates the model’s capability for accurate and explainable fault diagnosis.

#### Ablation experiments

**Figure 10:**
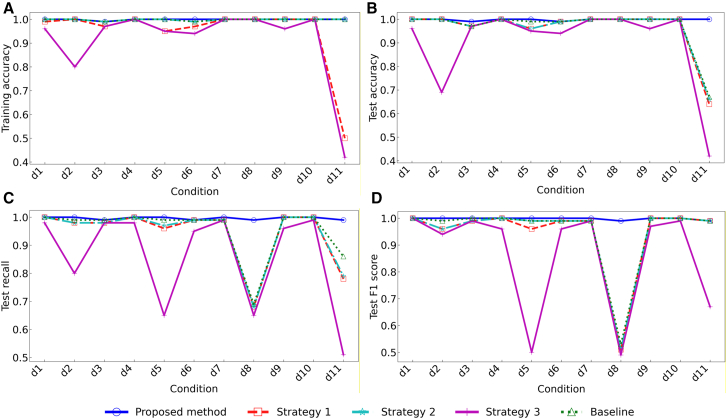
Diagnostic performance of the model in each condition (A) Comparison of training accuracy. (B) Comparison of test accuracy. (C) Comparison of test recall. (D) Comparison of test F1 score.

**Figure 11:**
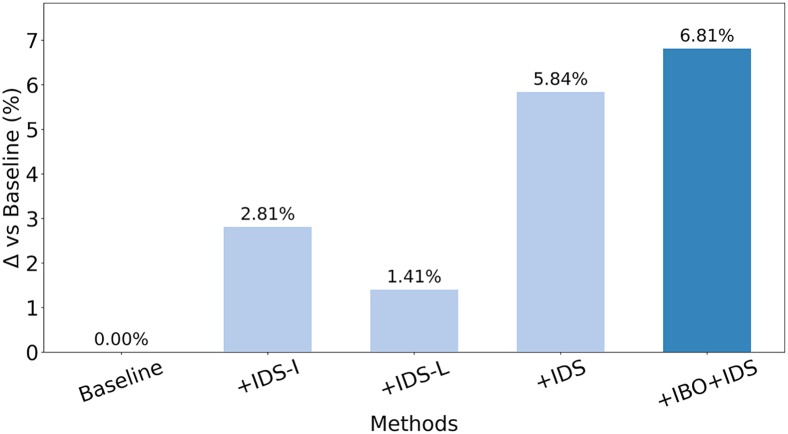
Percentage improvement of model accuracy by different modules

The effectiveness of each strategy in the proposed method was verified by using the dataset in group 2. The four strategies are proposed for ablation experiments. Baseline: Random forest. Strategy 1: Replace IDS with the D-S method based only on information improvement (IDS-I). Strategy 2: Replace IDS with the D-S method that is improved only based on local conflicts (IDS-L). Strategy 3: Remove the improved Bayesian optimization algorithm. The results indicate that the proposed method outperforms all the compared schemes, as shown in Table 6. It not only surpasses the classical machine learning baseline and other comparison strategies in key performance metrics such as diagnostic accuracy, recall, and F1 score, but also achieves the optimal overall performance. More importantly, by incorporating an improved Bayesian optimization algorithm, the method attains an effective balance between diagnostic performance and computational efficiency. Compared with Strategy 3, which exhibits similar performance, the proposed method significantly reduces training time while further improving diagnostic accuracy, demonstrating its practical potential and advancement. Furthermore, the classification performance of each strategy across different states is comprehensively evaluated using training accuracy, test accuracy, test recall, and test F1 score (Figure 10). The proposed method exhibits relatively stable accuracy across all states, whereas other strategies show substantial fluctuations. As shown in Figure 11, a bar chart illustrates the influence of different strategies on overall model accuracy. Compared with the baseline, the integration of IBO and IDS yields the most significant improvement, achieving an accuracy increase of 6.81%.

**Table 6:** Effect of different strategies on the diagnostic performance of the model

Method	Train time (s)	Train accuracy (%)	Test Accuracy (%)	Test recall (%)	Test F1 score (%)
Baseline	5.3	89.8	92.50	94.10	94.29
Strategy 1	9.2	97.00	95.10	94.84	95.06
Strategy 2	8.9	96.10	93.80	94.32	94.55
Strategy 3	15.2	98.50	97.90	98.74	99.74
Proposed method	10.1	99.23	98.80	99.73	99.74

### Comparative analysis

#### Comparison of different methods

The proposed method is inferior to the MGCN-TMIF-DST method in terms of training time consumed, but superior to the SDAE-BSD-DS method and DFCSSAES-WDS method. The experimental results are shown in Table 7. The proposed method introduces local decision tree information and conflicts, thereby increasing the computational time consumed. The SDAE-BSD-DS method outperforms the DFCSSAES-WDS method and the MGCN-TMIF-DST method in terms of metrics such as training set accuracy, test set accuracy, recall, and F1 scores. The SDAE-BSD-DS method leverages decision tree local information to enhance fault diagnosis accuracy. The proposed method outperforms the SDAE-BSD-DS method in terms of fault diagnosis performance. Compared with the SDAE-BSD-DS method, it improved the test set accuracy, recall, and F1 score by 1.1%, 0.1% and 0.1%, respectively. The IBO-RF-IDS method can effectively integrate local decision tree information and conflict processing to improve the accuracy of fault diagnosis.

**Table 7:** Performance comparison of different fault diagnosis methods

Method	Train time (s)	Train accuracy (%)	Test Accuracy (%)	Test recall (%)	Test F1 score (%)
Proposed method	10.1	99.30	98.67	98.68	98.72
DFCSSAES-WDS^a^	12.24	95.82	95.35	95.09	95.14
SDAE-BSD-DS^b^	14.6	97.12	96.46	98.55	98.59
MGCN-TMIF-DST^c^	15.21	92.66	91.72	93.31	93.21

a Gao et al.^16^

b Wang et al.^17^

c Zhang et al.^15^

**Figure 12:**
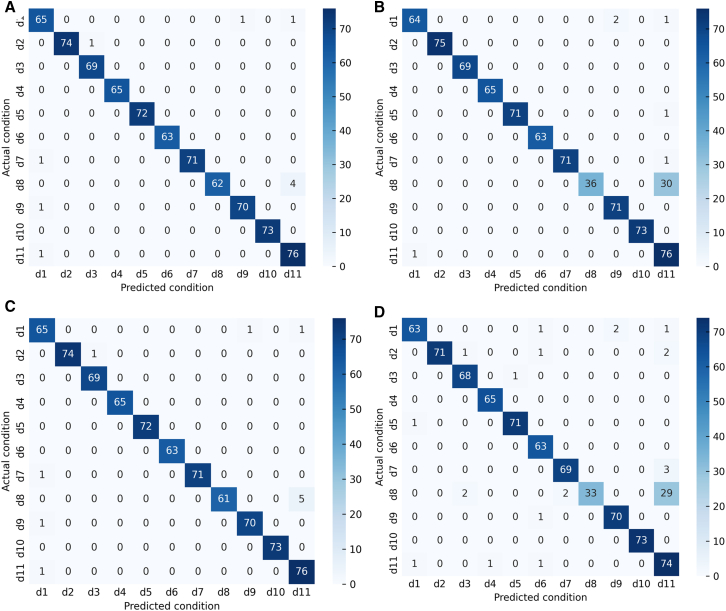
Confusion matrix diagrams of different fault diagnosis methods (A) Classification performance of the proposed method. (B) Classification performance of the DFCSSAES-WDS method. (C) Classification performance of the SDAE-BSD-DS method. (D) Classification performance of the MGCN-TMIF-DST method.

**Figure 13:**
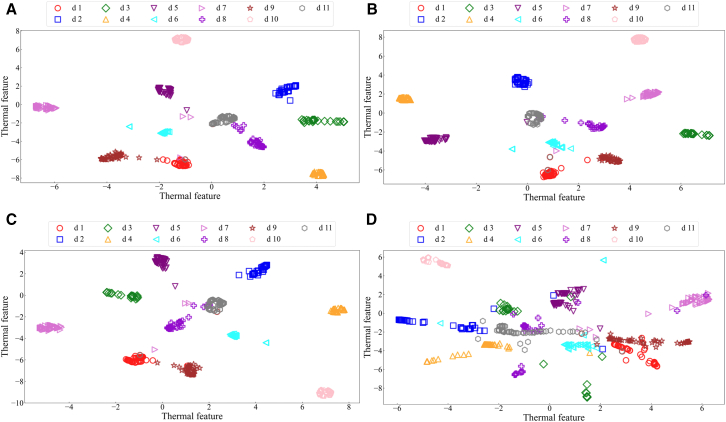
The *t*-SNE visual sample distribution of different methods (A) Feature clustering visualization of the proposed method. (B) Feature clustering visualization of the DFCSSAES-WDS method. (C) Feature clustering visualization of the SDAE-BSD-DS method. (D) Feature clustering visualization of the MGCN-TMIF-DST method.

**Figure 14:**
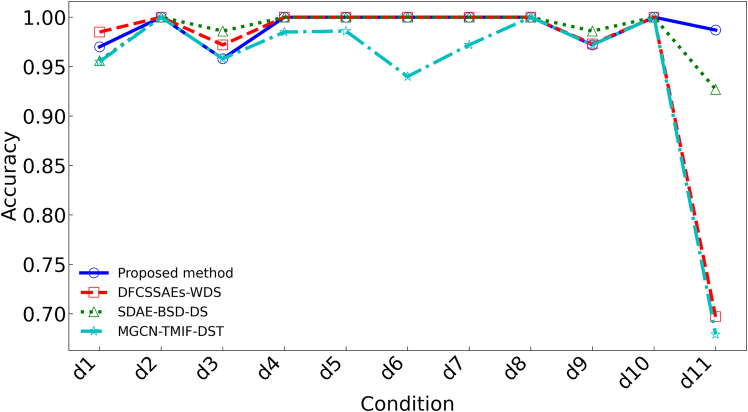
Diagnostic accuracy of the model in each condition

By analyzing the fault diagnosis confusion matrix diagram of the proposed method and three publicized methods (Figure 12). The experimental results show that the DFCSSAES-WDS method and the MGCN-TMIF-DST method have the largest diagnostic error at d8, with errors of (30/66) and (33/66), respectively. The primary reason is the failure to consider local decision tree conflicts and excessive reliance on prior knowledge. The SDAE-BSD-DS method exhibits a smaller diagnostic error on d8 compared to both the DFCSSAES-WDS method and the MGCN-TMIF-DST method. The SDAE-BSD-DS method can revise the decision tree BPA to reduce the influence of conflicting decision trees on the fusion result, but it lacks a reasonable weight distribution, resulting in an unreasonable fusion result. However, compared with the SDAE-BSD-DS method, the proposed method has the smallest diagnostic error on d8. The sample distribution for each condition diagnosis by different methods (Figure 13). In the diagnosis of each condition by the publicized method and the proposed method, the conditions d4, d6, and d10 have no intersection or overlap with other condition samples. Some samples in state d8 are misdiagnosed as state d11, which indicates that these methods misdiagnose condition d8. However, the number of sample intersections between conditions d8 and d11 diagnosed by the proposed method is small. The experimental results show the effectiveness of the proposed method. The classification accuracy of the above model in different conditions is shown in Figure 14. The diagnostic accuracy of the proposed method for condition d1 is lower than that of the DFCSSAES-WDS method. This is mainly because the DFCSSAES-WDS method has advantages in sample overlap processing. The diagnostic accuracy of the proposed method for condition d3 is lower than that of the SDAE-BSD-DS method; the main reason is that the SDAE-BSD-DS method has advantages in high-conflict transient faults. However, the proposed method performs better in balanced multi-source information fusion in terms of dealing with noise, conflict, and uncertainty. It has greater than 95% classification accuracy on conditions d4 to d11. It also demonstrated the effectiveness of introducing local decision trees for local conflicts and information.

The proposed method and the publicized methods are trained using a 10% sample of the training samples, as shown in Table 8. The proposed method is superior to the publicized methods in terms of training time and accuracy. The SDAE-BSD-DS method is superior to the DFCSSAES-WDS method and the MGCN-TMIF-DST method in terms of accuracy. SDAE effectively prevents overfitting through noise reduction ability under small samples, and BSD alleviates parameter conflicts by optimizing fusion weights. However, DFCSSAES-WDS is prone to performance degradation due to insufficient information under small samples. Compared with the SDAE-BSD-DS method, the proposed method improves 4.6%, 3.9%, 2.5%, and 2.5% in training accuracy, test accuracy, test set recall, and test set F1 score metrics, respectively. The SDAE-BSD-DS method relies too heavily on the induced variables. When domain knowledge is lacking, it is easy to fall into local optima, which leads to a bias in weight assignment. The proposed method takes advantage of dealing with uncertain information to improve the diagnostic performance. Experiments show that the proposed method can effectively diagnose diesel engine faults.

**Table 8:** Comparison of fault diagnosis performance of different models with small samples

Method	Train time (s)	Train accuracy (%)	Test Accuracy (%)	Test recall (%)	Test F1 score (%)
Proposed method	3.45	98.67	97.37	98.16	98.20
DFCSSAES-WDS^a^	5.67	93.45	92.60	93.95	93.91
SDAE-BSD-DS^b^	6.89	94.12	93.50	95.70	95.79
MGCN-TMIF-DST^c^	7.15	90.12	89.03	89.64	89.50

a Gao et al.^16^

b Wang et al.^17^

c Zhang et al.^15^

**Figure 15:**
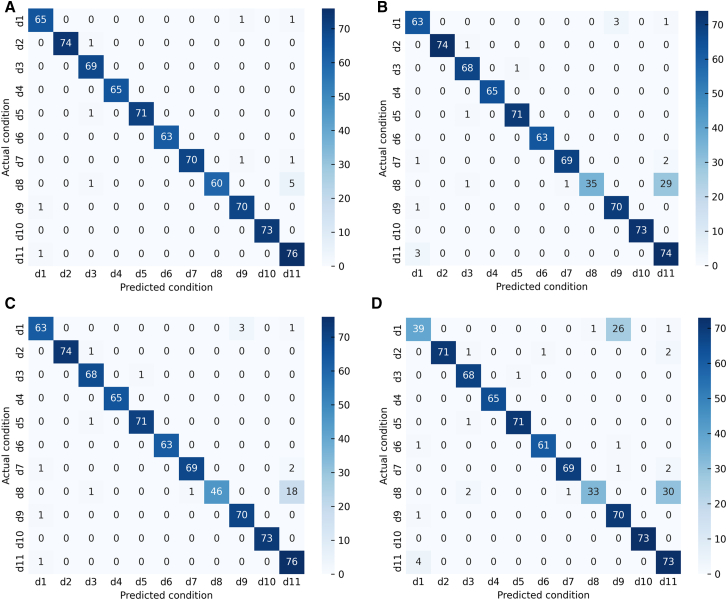
Confusion matrix of different models for fault diagnosis with small samples (A) Small-sample classification performance of the proposed method. (B) Small-sample classification performance of the DFCSSAES-WDS method. (C) Small-sample classification performance of the SDAE-BSD-DS method. (D) Small-sample classification performance of the MGCN-TMIF-DST method.

**Figure 16:**
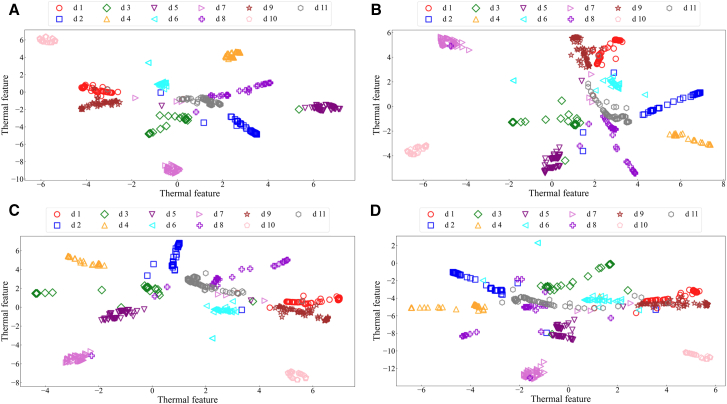
The *t*-SNE visual sample distribution of different methods under small samples (A) Small-sample feature distribution of the proposed method. (B) Small-sample feature distribution of the DFCSSAES-WDS method. (C) Small-sample feature distribution of the SDAE-BSD-DS method. (D) Small-sample feature distribution of the MGCN-TMIF-DST method.

**Figure 17:**
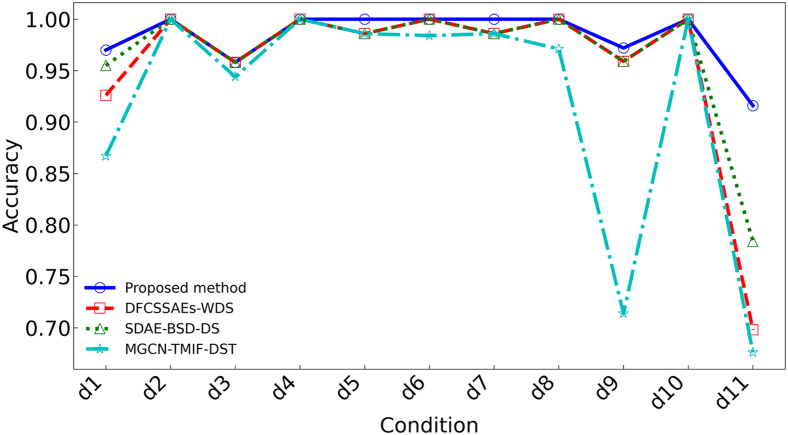
Diagnostic accuracy of the model in each condition

The confusion matrices of various models used for fault diagnosis with limited samples (Figure 15). As shown in Figures 15A–15C, the confusion matrix is presented. The number of correctly recognized samples by the proposed method for condition d8 is more than that by the publicized method. The main reason is that the publicized method relies solely on prior knowledge, which is insufficient for obtaining local prior knowledge from small samples. The proposed method introduces local information, enabling the model to comprehensively acquire knowledge and improve the recognition accuracy of condition d8. The diagnostic errors of the proposed method and the publicized method for different conditions are shown in Table 9. The above method has a very good recognition effect on conditions d4 and d10. Compared with the publicized method, the proposed method has fewer diagnostic errors for states d1 and d8, respectively. The sample distribution recognition by the proposed method and the publicized method for each condition, where part of the samples in condition d8 intersect with state d11 (Figure 16). But the proposed method has a small number of overlapping samples in these states. The DFCSSAES-WDS suffers from induced sorting bias and unstable weight assignment under small samples. The MGCN-TMIF-DST is deficient in handling information conflicts under small samples. This article enhances the accuracy of fault diagnosis by refining the combination rule based on both global and local information, as well as conflicts. As shown in Figure 17, the proposed method achieves more stable classification accuracy in small samples than the publicized methods. Compared with the full sample, the SDAE-BSD-DS method fluctuates considerably in terms of classification accuracy in small samples. However, the proposed method is more accurate than the SDAE-BSD-DS method for diagnosis. This is primarily because the SDAE-BSD-DS method does not account for the effects of introducing local conflicts. The experiments demonstrate that the proposed method is able to accurately recognize each condition under small samples.

**Table 9:** Different condition recognition errors for different models with small samples

	d1	d2	d3	d4	d5	d6	d7	d8	d9	d10	d11
Proposed method	65/67	74/75	69/70	0	71/72	0	70/72	60/66	70/71	0	76/77
DFCSSAES-WDS^a^	63/67	74/75	68/69	0	71/72	0	69/72	35/66	70/71	0	74/77
SDAE-BSD-DS^b^	63/67	74/75	68/69	0	71/72	0	69/72	46/66	70/71	0	76/77
MGCN-TMIF-DST^c^	39/67	71/75	68/69	0	71/72	61/63	69/72	33/66	70/71	0	73/77

a Gao et al.^16^

b Wang et al.^17^

c Zhang et al.^15^

**Figure 18:**
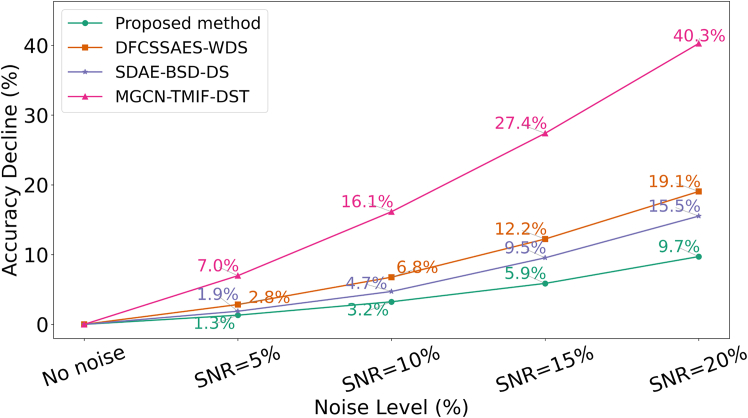
Decrease in the rate of model accuracy for different noise levels

The sample data collected in the actual industrial environment inevitably contains noise. This article examines the effects of different signal-to-noise ratios (SNRs) on the diagnostic performance of the model. The experiment results of different methods are shown in Table 10. When the SNR is between 0% and 5%, the SDAE-BSD-DS method has a lower accuracy rate in condition classification than the DFCSSAES-WDS method. Inversely, when the SNR is between 10% and 20%, the SDAE-BSD-DS method outperforms the DFCSSAES-WDS method in terms of accuracy and noise reduction. Their diagnostic accuracy surpasses that of the MGCN-TMIF-DST method in cases involving noise. The MGCN-TMIF-DST method does not incorporate an anti-noise design and is acutely sensitive to noise. The SDAE-BSD-DS method has been shown to suppress partial noise through information optimization. The DFCSSAES-WDS method enhances the noise immunity through the implementation of weighted fusion. The proposed method demonstrates superiority over the publicized method in terms of classification accuracy with SNR between 0% and 20%. The experimental results demonstrate that the proposed method is capable of maintaining high classification accuracy even under extreme noise. The outstanding robustness of the proposed method under high-noise conditions (Figure 18). The method consistently exhibits the lowest performance degradation across all SNR levels, and its performance advantage becomes increasingly pronounced as the noise level rises, highlighting its significant potential and value in complex and uncertain real-world industrial applications.

**Table 10:** Comparison of the diagnostic accuracy of models at different SNRs

Method	No noise	SNR = 5%	SNR = 10%	SNR = 15%	SNR = 20%
Proposed method	98.80	97.50	95.60	93.00	89.20
DFCSSAES-WDS^a^	96.85	94.10	90.30	85.00	78.40
SDAE-BSD-DS^b^	95.29	93.50	90.80	86.20	80.50
MGCN-TMIF-DST^c^	91.59	85.20	76.80	66.50	54.70

a Gao et al.^16^

b Wang et al.^17^

c Zhang et al.^15^

In summary, the developed IBO-RF-IDS outperforms DFCSSAES-WDS, SDAE-BSD-DS, and MGCN-TMIF-DST in terms of testing accuracy by margins of 3.36%, 2.24%, and 7.04% respectively. The efficacy of the IBO-RF-IDS method described herein for fault diagnosis under conditions of limited samples and noise has been empirically validated.

#### Benchmark test

The proposed method significantly outperforms three well-established machine learning algorithms (KNN, SVM, and RF) in overall performance, including prediction accuracy and F1 Score, thereby confirming its superiority, as shown in Table 11. Among the baselines, Random Forest (RF) exhibits the best performance and serves as the most competitive benchmark. Although the proposed method requires slightly longer training time, its superior accuracy demonstrates an effective trade-off between efficiency and performance. Overall, the benchmark results clearly verify the high efficiency and excellent performance of the proposed method, surpassing existing standard algorithms.

**Table 11:** Performance evaluation of the proposed method and the benchmark model

Method	Train time (s)	Train accuracy (%)	Test Accuracy (%)	Test recall (%)	Test F1 score (%)
Proposed method	10.02	99.31	98.65	97.88	98.54
KNN	0.5	91.24	89.46	89.48	90.54
SVM	15.6	93.36	92.44	92.47	92.41
RF	5.3	95.66	94.50	94.44	94.41

#### Comparison of different optimization algorithms

As shown in Table 12, the IBO algorithm decisively outperforms other classic optimization algorithms like the Particle Swarm Optimization (PSO), Genetic Algorithm (GA), and Standard Bayesian Optimization (BO). In terms of computational cost, IBO is remarkably efficient, with a training time of only 10.08 s, underscoring its rapid convergence speed. Beyond efficiency, IBO also achieves top-tier performance, delivering a test accuracy of 98.69%, an F1-score of 98.68%, and a recall rate of 97.90%. Such outstanding results on the test set prove that the model possesses strong generalization capabilities. In conclusion, IBO is a highly effective optimization method, uniquely combining speed with exceptional performance.

**Table 12:** Effect of optimization algorithms on model performance

Method	Train time (s)	Train accuracy (%)	Test Accuracy (%)	Test recall (%)	Test F1 score (%)
IBO	10.08	99.43	98.69	97.90	98.68
PSO	15.8	99.15	98.31	97.25	98.20
GA	32.45	99.32	98.51	97.56	98.43
BO	19.82	99.28	98.47	97.51	98.35

#### Decision tree fusion using different combination rules

**Figure 19:**
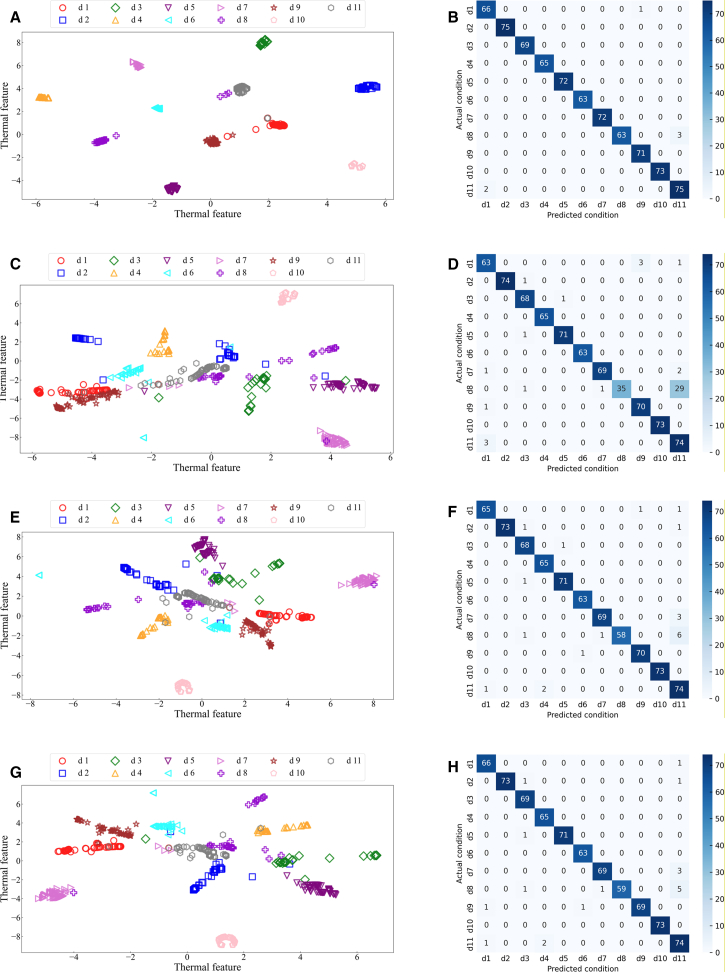
The *t*-SNE and confusion matrix diagrams of the decision tree combination rules methods (A and B) are the feature distribution and confusion matrix of the IDS. (C and D) are the feature distribution and confusion matrix of the classical D-S theory combination rule. (E and F) are the feature distribution and confusion matrix of the weighted combination rule. (G and H) are the feature distribution and confusion matrix of the BSD hybrid combination rule.

This article compares the IDS method with three different decision tree combination rule methods. The t-SNE visualization and confusion matrix of these methods are presented in Figure 19. Method 1: Classical D-S theory combination rule.^15^ Method 2: Weighted combination rule.^16^ Method 3: BSD hybrid combination rule.^17^ The IDS method has a good classification effect on each state; the gap between the condition category clusters is obvious, and the sample overlap is small (Figure 19A). As shown in Figure 19B, three samples in condition d8 were misdiagnosed as condition d11. The IDS method considers both global and local information, as well as conflict processing. It uses Shannon entropy combined with Jousselme distance to provide dynamic weights for decision trees. This enhances the model’s ability to fuse multi-source information, allowing it to focus more on the decision tree information in each condition. However, the sample distribution in each condition recognition by the publicized method is very scattered, and there are a large number of samples that intersect (Figure 19C). For example, 29 samples in condition d8 were misdiagnosed as condition d11 (Figure 19D). Method 1 overlooks the local decision tree conflict and the amount of information, potentially leading the model to dominate the normal condition and compromise the accuracy of fault condition diagnosis. Conditions d7 and d8 intersect with the samples in condition d11, respectively (Figure 19E). Three samples in condition d7 and 6 samples in condition d8 are misdiagnosed as condition d11 (Figure 19F). Method 2 struggles with handling local decision tree conflicts. Conditions d7 and d8 intersect with the samples in condition d11, respectively (Figure 19G). Three samples in condition d7 and five samples in condition d8 are misdiagnosed as d11 (Figure 19H). Method 3 is a static fusion method, which cannot fully mine the important information between decision trees. By contrast, the proposed IDS method incorporates the enhanced D-S theory at the tree-level decision fusion stage of random forests, introducing a combined metric of Shannon entropy and Jousselme distance to jointly quantify the uncertainty and conflict in the outputs of each decision tree, enabling adaptive trusted weighted fusion. This strategy not only refines traditional D-S fusion at the evidence layer but also improves the robustness and credibility of the random forest model itself, thereby clearly distinguishing it from previously proposed D-S-based methods.

The results of the above three methods on metrics such as training time are shown in Table 13. In terms of training time consumption, the IDS method is better than Method 2 and Method 3, which are longer than Method 1. Since it introduces Shannon entropy weighted Jousselme distance to improve the decision tree combination rule, considering local decision tree information and conflicts, it increases the training time. The proposed IDS method outperforms method 1 in all three key metrics: accuracy, recall, and F1 score. Among the publicized methods, method 3 has better diagnostic performance than methods 1 and 2. Method 3 has poor explainability and requires a large number of samples for model training, which leads to low diagnostic accuracy. Method 1 does not incorporate a weighting operation. In Method 2, the Bayesian method relies on the prior distribution assumption, and the performance decreases when the data is insufficient. Compared to method 3, the proposed IDS method improves 2.9%, 3.0%, 1.7%, and 1.7% in the metrics of training accuracy, testing accuracy, testing recall, and testing F1 score, respectively. The Shannon entropy-based weighted Jousselme distance can dynamically assign weights to the decision trees. The decision tree combination rule is to be improved on the basis of local conflict and information. Furthermore, this approach enhanced the ability of multi-source information fusion. The experiments demonstrate the effectiveness of the IDS method for diesel engine fault diagnosis.

**Table 13:** Performance comparison of different combination rule methods

Method	Train time (s)	Train accuracy (%)	Test Accuracy (%)	Test recall (%)	Test F1 score (%)
IDS method	10.3	99.90	99.45	99.21	99.23
Method 1^c^	5.6	93.21	92.63	93.95	93.91
Method 2^a^	12.2	95.6	94.38	97.27	97.32
Method 3^b^	15.6	97	96.43	97.54	97.58

a Gao et al.^16^

b Wang et al.^17^

c Zhang et al.^15^

### Limitations of the study

This study is limited to specific load conditions and simulated application scenarios of marine diesel engines, within which the proposed approach achieves reliable diagnostic performance. Its applicability and robustness under broader real-world operating environments have yet to be fully assessed. In addition, potential factors such as sensor noise, ambient variability, and differences in engine configuration were not considered. It is therefore recommended that future studies validate the approach using large-scale real fault datasets and explore its extension toward a lightweight and generalizable diagnostic framework for practical marine applications.

## Resource availability

### Lead contact

Requests for further information and resources should be directed to and will be fulfilled by the lead contact, Baozhu Jia (jiabzh@gdou.edu.cn).

### Materials availability

This study did not generate new unique reagents.

### Data and code availability

• Requests for diesel engine thermal parameter data used in this work should be directed to the lead contact.
• All original code has been deposited on GitHub and is publicly available at https://github.com/xiezaimi-png/Thermal-fault-diagnosis-of-diesel-engines.git as of the date of publication.
• Any additional information required to reanalyze the data reported in this article is available from the lead contact upon request.

## Acknowledgments

This work was supported by the 10.13039/501100001809National Natural Science Foundation of China (Grant no. 52071090, 52401418, 52201355) and the Guangdong Province Overseas Famous Teacher Project (No. MS202500036).

## Author contributions

Conceptualization, C.M.M.; methodology, Z.M.X; investigation, Z.M.X., and C.M.M.; writing -– original draft, Z.M.X.; writing – review and editing, Z.M.X., C.M.M., and B.Z.J.; funding acquisition, B.Z.J.; resources, B.Z.J. and Z.M.X.; supervision, Z.M.X., C.M.M., and B.Z.J.

## Declaration of interests

We declare no competing interests related to this study.

## STAR★Methods

### Key resources table

**Table:** 

REAGENT or RESOURCE	SOURCE	IDENTIFIER
**Software and algorithms**		

Python 3.7	Python Software Foundation	https://www.python.org/
PyTorch 2.11.0	Linux Foundation	https://pytorch.org/
Ansys Workbench 2020	Ansys Software Foundation	https://www.ansys.com/zh-cn/products/ansys-workbench

**Other**		

Diesel engine partial dataset	This paper	https://github.com/xiezaimi-png/Thermal-fault-diagnosis-of-diesel-engines.git

### Experimental model and study participant details

This study did not involve biological experimental models or human participants. Normal-condition data were collected from an actual marine diesel engine, and fault-condition data were generated using a one-dimensional thermodynamic simulation model. Biological attributes (species, genotype, age, sex/gender) and ethical approval requirements are therefore not applicable.

### Method details

#### Generalized multi-source information fusion method

##### Parameter pre-processing and screening

The multi-source data used in this paper include a variety of thermodynamic parameters collected by various subsystems of the diesel engine: intake, cooling, combustion, and exhaust. The relevant concepts of the Pearson correlation coefficient and mutual information are defined, and the screening method of thermal parameters is proposed on this basis. This method can reduce redundant parameters to improve the computational speed and diagnostic performance of the model. The adopted diesel engine thermal parameters and their symbolic representations are shown in Table S1.

The parameter matrix $X\in R^{m\times n}$ is given by (Equation 1).

(Equation 1) $$X=\left(\begin{array}{ccc} x_{11} & \ldots & x_{1n}\\ \vdots & \ddots & \vdots \\ x_{m1} & \cdots & x_{mn} \end{array}\right)$$

Where ***X*** is the original data matrix, *m* is the number of samples, and *n* is the number of features for each sample.

The data ***X*** was input to the Pearson correlation coefficient^21^ of equation (Equation 2) to obtain the low-correlation parameter ***Z***, as shown in (Equation 3).

(Equation 2) $$C=XEE^{T}$$

(Equation 3) $$Z=\left\{j|\forall k\in \left\{1,2,\ldots ,p\right\}\left|C_{jk}\right|<\theta \right\}$$

where $E\in R^{n\times p}$ are the score matrix and projection matrix of the principal components, respectively.

The data *X* was input into equation (Equation 4)^22^ to obtain the parameters' mutual information values. The target categories ***Y*** exceeded a threshold value *γ* of 0.95, as shown in (Equation 5).

(Equation 4) $$M\left(X^{\prime },Y\right)=\iint d_{x}d_{y}\vartheta \left(x,y\right)\mathrm{lg}\frac{\vartheta \left(x,y\right)}{\vartheta \left(x\right)\vartheta \left(y\right)}$$

(Equation 5) $$I=\left\{j|M\left(X_{:,j};Y\right)\geq \gamma \right\}$$

where *ϑ*(*x*) and *ϑ*(*y*) are the edge density functions of ***X*** and ***Y***, respectively.

Pearson’s correlation coefficient was screened to a 4-dimensional parameter matrix ***Z***. The mutual information was screened to an 8-dimensional parameter matrix ***I***. They are concatenated to a 12-dimensional parameter matrix ***X***″. It can be presented as

(Equation 6) $$X^{\prime }=Z\cup I$$

##### Random forest

Accordingly, the use of Random Forest^23^ (RF) has the advantage of dealing with high dimensionality and adapting to nonlinearly noisy data. The decision tree’s randomness in each class and the decision path’s backward fault logic are combined to focus on the different conditions of key parameters. The *t*th decision tree *h*_*t*_ by bootstrap from *X*′ sampling, each node is randomly to select sqrt(*m*) part of the features, leaf nodes as the output, the results ***G***(*x*) can be presented as

(Equation 7) $$G\left(c|x\right)=\frac{1}{T}\sum_{t=1}^{T}p_{t}\left(c|x\right),x\in X^{\prime }$$

where *T* is the total number of decision trees; ***Y*** is the set of all categories; *p*_*t*_(*c*|*x*) is the probability that the *t*th decision tree predicts that sample *x* belongs to category *c*; *c*∈***Y***; *G*(*c*|*x*) are the sum of the decision probabilities for all decision trees {*DT*_1_,*DT*_2_,⋯,*DT*_*m*_} in category *c*.

In the classification framework of random forests, Gini index and entropy-based information gain metrics are usually used for feature selection. In this study, the Gini coefficient is used as the main feature selection standard. The Gini index, which ranges from 0 to 1, is used as an inverse indicator of feature importance. “0” indicates the maximum information gain (indicating the most significant parameter). “1” indicates the minimum information gain (indicating the least significant parameter). The mathematical expression for the Gini coefficient is

(Equation 8) $$Gini\left(D\right)=1-\sum_{i=1}^{11}G_{i}^{2}$$

where *G*_*i*_ is the probability that category *i* is in the dataset. *D* represents the sample subset of the current node in the random forest.

##### Improved Bayesian optimization for RF tuning

Hyperparameter optimization can enhance both the computational efficiency and accuracy of the RF model. The Bayesian optimization algorithm^12^ (BO) is particularly advantageous due to its capability to effectively address global hyperparameter optimization in highly nonlinear scenarios. In contrast, traditional models typically employ an acquisition function based on the Expectation Improvement Criterion (EI) to tackle constrained problems. In this paper, we introduce an innovative approach by implementing Penalization Constrained Optimization EI (PCEI). This method ensures that sample points failing to meet constraints are positioned far from the infeasible region. For a detailed explanation of the improved EI calculation incorporating penalty considerations, please refer to equation (Equation 9).

(Equation 9) $$EI\left(x\right)=\left(f\left(x^{+}\right)-\mu \left(x\right)\right)\Psi \left(\frac{f\left(x^{+}\right)-\mu \left(x\right)}{\sigma \left(x\right)}\right)+\sigma \left(x\right)\psi \left(\frac{f\left(x^{+}\right)-\mu \left(x\right)}{\sigma \left(x\right)}\right)-Z$$

where *EI*(*x*) represents the expected acquisition function; *x*^+^ denotes the current best consistent input. Specifically, the number of decision trees, the maximum depth, the maximum number of features, and the minimum number of samples at leaf nodes. *f*(*x*^+^) refers to the currently observed optimal value of the objective function; and *μ*(*x*) and *σ*(*x*) signify the predicted mean and standard deviation of the surrogate model at sample point *x*, respectively. Furthermore, Ψ(·) and *ψ*(·) represent the cumulative distribution function and probability density function of the standard normal distribution, respectively.

(Equation 10) $$Z=\left\{ \begin{array}{l} 0,u_{i}\left(x\right)\leq 0,i=1,2,\cdots ,N\\ C,\text{Other} \end{array}\right.$$

where *u*_*i*_(*x*) represents the predicted value of the *i*th constraint derived from the predictive distribution model for the current sample point *x*, and *C* denotes a larger threshold. In equation (Equation 10), when *u*_*i*_(*x*)≤0 indicates that the current sample point satisfies the constraint, Z is set to 0 at this moment, signifying that no penalty is applied to the PCEI criterion. Conversely, if it is determined that the current sample point does not meet the constraint, Z is assigned a value of *C*. Furthermore, by integrating Gaussian processes^24^ with the PCEI criterion, this paper develops the IBO algorithm; its specific process is outlined as follows.

(1) Initially, sample points are generated randomly, and a predictive distribution model of the objective function is established.
(2) Assess whether the new sample points satisfy the constraints; identify the new sample points corresponding to the *k*th iteration, and update the dataset accordingly.
(3) Proceed to iterate for the (*k*+1)th time, repeating steps 1 and 2 until either the maximum number of 30 iterations is reached or the tolerance error falls below the maximum allowable error threshold. At this point, terminate the iteration process and present the optimal result.

##### Decision tree feature selection

SHAP has been widely used to enhance model explainability.^25^ The traditional computation of SHAP necessitates a comprehensive exploration of all potential parameter combinations, leading to considerable computational overhead and an exponential increase in complexity that escalates with the dimensionality of the parameters. Tree SHAP^26^ structure obviates the need for exhaustive enumeration of all parameter combinations, thereby resulting in a substantial enhancement in computational efficiency. In the process of Tree SHAP value calculation, the concept of contribution value is used to quantitatively characterize the influence of each thermal parameter on the model prediction results. The structural diagram is presented in Figure S1. The SHAP value *ζ*_*i*_ can be computed using equation (Equation 11).

(Equation 11) $$\zeta_{i}=\sum_{S\in P\setminus \left\{i\right\}}\frac{\left|S\right|!\left(u-\left|S\right|-1\right)!}{u!}\left(f\left(S\cup \left\{i\right\}\right)-f\left(S\right)\right)$$

where *P* is the set of all parameters; *S* is a subset of the parameters; *P*/{*i*}encompasses all feasible parameter combinations excluding the target parameter *i*; *u* denotes the number of parameters in the subset *S*.; *f*(*S*) is the contribution generated by the set *S*; *f*(*S*∪{*i*}) is the contribution when parameter *i* is added.

#### IBO-RF-IDS method

Classical D-S theory^27^ is one of the techniques in the field of information fusion. Its main advantage is to solve the problem of uncertainty. The IBO-RF method provides the BPA based on a decision tree Γ_*i*_(*A*) for the D-S theory, so as to realize the reliability evaluation of results and multi-source information fusion, and improve the accuracy of fault diagnosis. It conforms to the requirements of equation (Equation 12).

(Equation 12) $$\left\{ \begin{array}{l} \Gamma_{i}\left(A\right)=0,A=\emptyset \\ \sum_{A\subseteq \Theta }\Gamma_{i}\left(A\right)=1,A\neq \emptyset \end{array}\right.$$

where Θ is the set of all conditions, 2^Θ^∈[0,1], ∅ represents the empty set.

The traditional D-S theory reduces the uncertainty of decision tree fusion results, leading to a more reliable BPA. The base probability Γ″(*A*) after fusion are calculated as

(Equation 13) $$\Gamma^{\prime }\left(A\right)=\sum_{A_{1}\cap A_{2}\cap \cdots \cap A_{n=}A}\prod_{i=1}^{n}\Gamma_{i}\left(A_{i}\right)/\left(1-K\right)$$

where the set of conditions {*A*_1_,*A*_2_,⋯,*A*_*n*_}∈Θ; Γ′(*A*) is the sum of the products of the BPAs of the decision trees whose intersection is not the empty set in the subset of decision generics; *K* is the degree of conflict between the decision trees.

Although the traditional Dempster’s combination rule can deal with decision conflicts, it uniformly normalizes all conflicts into the decision tree BPA, which results in some information being ignored. At the same time, existing evidence combination methods often fail to fully consider the complexity of the evidence structure and their sensitivity to non-specific focus elements. To this end, this study introduces Shannon entropy^28^ and Jousselme distance^20^ to calculate the dynamic weight of the focus element in the decision tree, so as to improve the evidence combination rule: part of the conflict is reassigned to the decision tree BPA, and the rest of the conflict is used as the credibility measure of the decision result. This method considers the amount of information and the conflict relationship between focus elements, thereby making the fusion result more accurate and avoiding information loss. Based on this, this paper improves the unnormalized base belief Γ″(*A*) and conflict *K*, and its calculation is defined in (Equation 14).

(Equation 14) $$\left\{ \begin{array}{l} \Gamma^{\prime \prime }\left(A\right)=\left(1-K\right)\Gamma^{\prime }\left(A\right)\\ K=\sum_{A_{1}\cap A_{2}\cap \cdots \cap A_{n=}\emptyset }\prod_{i=1}^{n}\Gamma_{i}\left(A_{i}\right)) \end{array}\right.$$

For each body of evidence, the Pignistic probability *P*_*i*_(*A*) and average probability distribution *P*_*avg*_ of any element *A* in the identification frame Θ can be calculated as shown in (Equation 15).

(Equation 15) $$\left\{ \begin{array}{l} P_{i}\left(A\right)=\sum_{A\in \Theta }\frac{\Gamma_{i}\left(A\right)}{\left|A\right|}\\ P_{avg}\left(A\right)=\frac{1}{n}\sum_{i=1}^{n}P_{i}\left(A\right) \end{array}\right.$$

where Γ_*i*_(*A*) denotes the probability that element A is in the *i*th decision tree, |*A*| is the number of subsets containing element *A*, and n is the total number of decision trees. The sum of the subset of classes in which element *A* belongs is defined as *P*_*i*_(*A*). Averaging over each decision tree with element *A* gives *P*_*avg*_(*A*). The Jousselme distance (JS) between element A and its mean in the *i*th decision tree is further calculated, denoted *J*_*i*_(*A*). Its calculation formula is given in Equation 16.

(Equation 16) $$J_{i}\left(A\right)=\frac{1}{\left|A\right|}\sum_{A\in \Theta }^{\left|A\right|}\sqrt{\frac{1}{2}{\left(P_{i}\left(A\right)-P_{avg}\left(A\right)\right)}^{T}D\left(P_{i}\left(A\right)-P_{avg}\left(A\right)\right)}J\left(P_{i}\left(A\right),P_{avg}\left(A\right)\right)\in \left[0,1\right]$$

where *J*(*P*_*i*_(*A*),*P*_*avg*_(*A*)) is the similarity distance between *P*_*i*_(*A*) and *P*_*avg*_(*A*) as the local information between the decision trees; *P*_*i*_(*A*) and *P*_*avg*_(*A*) are vector forms of 2^*n*^×1; *D* is the matrix form of 2^*n*^×2^*n*^; $D\left(i,j\right)=\frac{\left|E_{i}\cap E_{j}\right|}{\left|E_{i}\cup E_{j}\right|}$; ∀*E*_*i*_,*E*_*j*_∈*P*(*A*); *E*_*i*_ and *E*_*j*_ are assumptions on *P*_*i*_(*A*) and *P*_*avg*_(*A*), respectively.

To quantify the amount of information ϒ_*i*_(*A*) carried by the subset containing element *A* in each decision tree, the calculation formula is given in (Equation 17).

(Equation 17) $$\left\{ \begin{array}{l} {ϒ}_{i}\left(A\right)=-\sum_{A\in \Theta }P_{i}\left(A\right)/\log_{2}\left(P_{i}\left(A\right)\right)\\ H_{i}\left(A\right)=\frac{e^{-{ϒ}_{i}\left(A\right)}}{\sum_{j=1}^{\left|A\right|}e^{-ϒj\left(A\right)}} \end{array}\right.$$

where *H*_*i*_(*A*) represents the weight of the *i*th decision tree, calculated using the Softmax reverse entropy method, which measures the amount of information carried by the decision tree.

The Shannon entropy weighted Jousselme distance *s*_*i*_(*A*) are calculated

(Equation 18) $$s_{i}\left(A\right)=\lambda \times J_{i}\left(A\right)+\left(1-\lambda \right)\times H_{i}\left(A\right)$$

where *λ* is the weight coefficient used to balance the local and global information amount in the *i*th decision tree, with its value set to 0.5.

The local conflict ∂_*ij*_(*A*) between element *A*_1_ and element *A*_2_ in the *i*th decision tree and the *j*th decision tree is calculated as follows:

(Equation 19) $$\left\{ \begin{array}{l} \partial_{ij}\left(A\right)=\sum_{A=A_{1}\cap A_{2}}\Gamma_{i}\left(A_{1}\right)\Gamma_{j}\left(A_{2}\right),A=\emptyset \\ k\left(A\right)=\frac{2}{n\left(n-1\right)}\sum_{i<j}\partial_{ij}\left(A\right)\\ \eta \left(A\right)=e^{-2.5k\left(A\right)} \end{array}\right.$$

where ∂_*ij*_(*A*) is the local conflict between decision tree *i* and decision tree *j*. *i*<*j* is the lower half of the symmetry of the *i*th decision tree with the *j*th decision tree to avoid computational duplication. *A*_1_,*A*_2_∈Θ. *k* represents the average value of local conflicts, and based on this, the exponential function is used to construct the conflict discount coefficient *η*.

In Equation 20, when the decision tree conflict *K* is small, *K* tends to 0, the combination result is similar to the classical D-S theory combination result, which is determined by Γ″(*A*). When the decision tree conflict *K* is large, *K* tends to 1, and the combination result is determined by *ηξ*(*A*). Γ″(*A*) and *K*′(*A*) are the decision tree fused BPA and conflict, respectively.

(Equation 20) $$\left\{ \begin{array}{l} \Gamma^{\prime \prime \prime }\left(A\right)=\Gamma^{\prime \prime }\left(A\right)+K\eta \left(A\right)s\left(A\right),A\neq \emptyset \\ K^{\prime }\left(A\right)=K\left(1-\eta \left(A\right)\right),A=\emptyset \end{array}\right.$$

where *s* and *η* represent the weight value of local conflict and the amount of information, respectively. Based on this, part of the conflict is assigned to the event BPA, and the rest is used as the reference for the result credibility to measure the reliability of the fusion result.

The BPA ∂ obtained by fusing all decision trees is applied to Equation 21 to obtain the output label *y*_pre_

(Equation 21) $$y_{\text{pre}}=\arg\;\max_{m}\;\partial$$

#### Overall framework

This paper clearly illustrates the proposed marine diesel engine intelligent fault diagnosis method based on generalized multi-source information fusion. The process consists of four primary steps: parameter preprocessing and screening, hyperparameter optimization, model interpretable feature selection, multi-source information fusion, and fault diagnosis. The main steps are depicted in Figure S2.

Step 1: The multi-source samples are obtained by using the simulation model calibrated on the actual machine. The Pearson correlation coefficient and mutual information are used to screen the important parameters with low correlation and influence on the target condition. It can improve the computational efficiency of the model and enhance the parameter explainability.

Step 2: The random forest hyperparameters are automatically optimized by the improved Bayesian algorithm to accelerate the convergence of the model. The IBO-RF is initialized based on the optimal hyperparameters. The contribution of each parameter to the condition is explained using Tree SHAP. Parameters with higher SHAP values are preferentially selected for retraining to enhance the model’s robustness.

Step 3: A generalized multi-source information fusion method based on IBO-RF-IDS is proposed. The Shannon entropy weighted Jousselme distance is used to measure the information content of each focal element in the decision tree, and it is used to improve the evidence combination rules to avoid the loss of part of the internal information. In addition, the IBO-RF-IDS method can improve the accuracy of fusion result and make the predicted labels of fault samples highly consistent with the actual labels.

Step 4: Obtain the decision tree fused BPA from the IBO-RF-IDS method and transform it into predictive labels to achieve diesel engine thermal fault diagnosis.

##### Data acquisition

The dataset employed in this study was collected from a marine pasture vessel operating in the Liushawan waters of Leizhou City, Zhanjiang, Guangdong, China, which is equipped with an L6160ZLCZ-89 diesel engine. A total of 24 thermal parameters (detailed in Table S1) were selected to facilitate the diagnosis of 12 representative engine operating states (detailed in Table S2). The definition of these states is primarily informed by the professional maintenance manual in combination with on-site investigations conducted at the shipyard enterprise. The system records raw data every 6 seconds and saves it in CSV files, forming the sample data set of the marine diesel engine operating time series. The raw data undergoes rigorous pre-processing, including elimination of blanks and duplicate records, interpolation of missing values, as well as anomaly detection and correction through a sliding window algorithm. Based on the ship’s design parameters and actual operational data, a one-dimensional thermal simulation model of the diesel engine was developed. The design parameters of the diesel engine are shown in Table S3. To validate its accuracy, the simulation results were compared with measurements collected from the vessel. The comparison indicates that the relative errors of all key performance parameters remain within 5%. These results demonstrating that the model possesses a high level of fidelity. Overall, the validity of the established one-dimensional thermodynamic model is verified by the data collected from a real ship. The detailed results are presented in Figure S3. The variations of these parameters under different fault conditions are illustrated in Figures S4A–S4F, where the horizontal axis denotes the crank angle and the vertical axis represents the parameter amplitudes. The combined dataset, integrating both simulation and real-ship data, was subsequently normalized and partitioned into training (70%) and testing (30%) sets. These datasets were employed to evaluate performance metrics, including model effectiveness and prediction accuracy.

##### Experimental setup

The superiority of the fault diagnosis method based on IBO-RF-IDS was verified by using the dataset in group 3. The model is implemented in Python 3.7. using scikit-learn. Training and testing of the networks are performed on a laptop with Windows 11 operating system, an AMD Ryzen 7 6800H CPU with Radeon Graphics, and an NVIDIA GeForce RTX 3060 6 GB Laptop GPU. The test data set is used as input to the proposed method, the Decision Fusion of Class-Specific Stacked Autoencoder Combines with Weighted Dempster-Shafer (DFCSSAEs-WDS) method,^16^ a Tacked Denoising Autoencoder combines with Belief Sinkhorn Distance and Dempster-Shafer Evidence Theory (SDAE-BSD-DS) method,^17^ and a Modified Graph Convolution Network-Trusted Multi-Source Information Fusion Combines with the Reduced D-S Theory (MGCN-TMIF-DST) method^15^ to train the four methods, respectively. The parameter settings for the four methods are shown in Table S4. The proposed methods are evaluated for diagnostic performance by the metrics of training time, accuracy, recall, and F1 score.^18^ These metrics are defined in equations (Equations 22, 23, 24, and 25).

(Equation 22) $$Precision=\frac{y_{\text{true}}}{y_{\text{true}}+y_{\text{false}}}\times 100\%$$

(Equation 23) $$Recall=\frac{y_{\text{true}}}{y_{\text{true}}+y_{\text{none}}}\times 100\%$$

(Equation 24) $$F1=\frac{2\times Precision\times Recall}{Precision+Recal}\times 100\%$$

(Equation 25) $$\text{Accuracy}=\frac{y_{\text{true}}+y_{\text{tn}}}{y_{\text{true}}+y_{\text{false}}+y_{\text{none}}+y_{\text{tn}}}\times 100\%$$

where *y*_true_ is the number of samples correctly diagnosed by the model (True Positives), *y*_fasle_ is the number of samples incorrectly identified as faults (False Positives), and *y*_none_ is the number of faulty samples missed by the model (False Negatives), *y*_tn_ is the number of samples correctly identified as normal (True Negatives).

### Quantification and statistical analysis

Statistical analysis and machine learning workflows were implemented using Python 3.7 with standard data processing libraries, including NumPy, Pandas, SciPy, and scikit-learn. The study utilized a dataset of 2,567 samples generated through one-dimensional thermodynamic simulations based on a GT-Power model, representing eleven distinct health states with approximately 233 samples per state. To optimize the feature space, the dimensionality of diesel engine thermal parameters was reduced and their interpretability improved by calculating the Pearson correlation coefficient and mutual information, with correlation coefficients exceeding 0.9 considered indicative of strong inter-parameter correlation. Following this preliminary screening, SHAP values were computed to quantify feature contributions. The features were randomly partitioned into training (70%) and testing (30%) sets using stratified sampling. Model performance was quantified using a Random Forest classifier with hyperparameters optimized via an improved Bayesian optimization algorithm. Additionally, IDS-based decision tree fusion was applied. Evaluation was performed using five-fold cross-validation, with accuracy, recall, and F1-score reported as mean ± standard deviation.
