# Supplementary material for: Marine diesel engine reliable intelligent fault diagnosis method based on the generalized multi-source information fusion
Source: iScience. 2025 Dec 5;29(1):114345. doi: 10.1016/j.isci.2025.114345 (PMC12811478; doi:10.1016/j.isci.2025.114345)
Supplement: Document S1. Figures S1–S4 and Tables S1–S4 [file mmc1.pdf]

**iScience, Volume *29***

## Supplemental information

# Marine diesel engine reliable intelligent fault diagnosis method based on the generalized multi-source information fusion

**Zaimi Xie, Chunmei Mo, and Baozhu Jia**

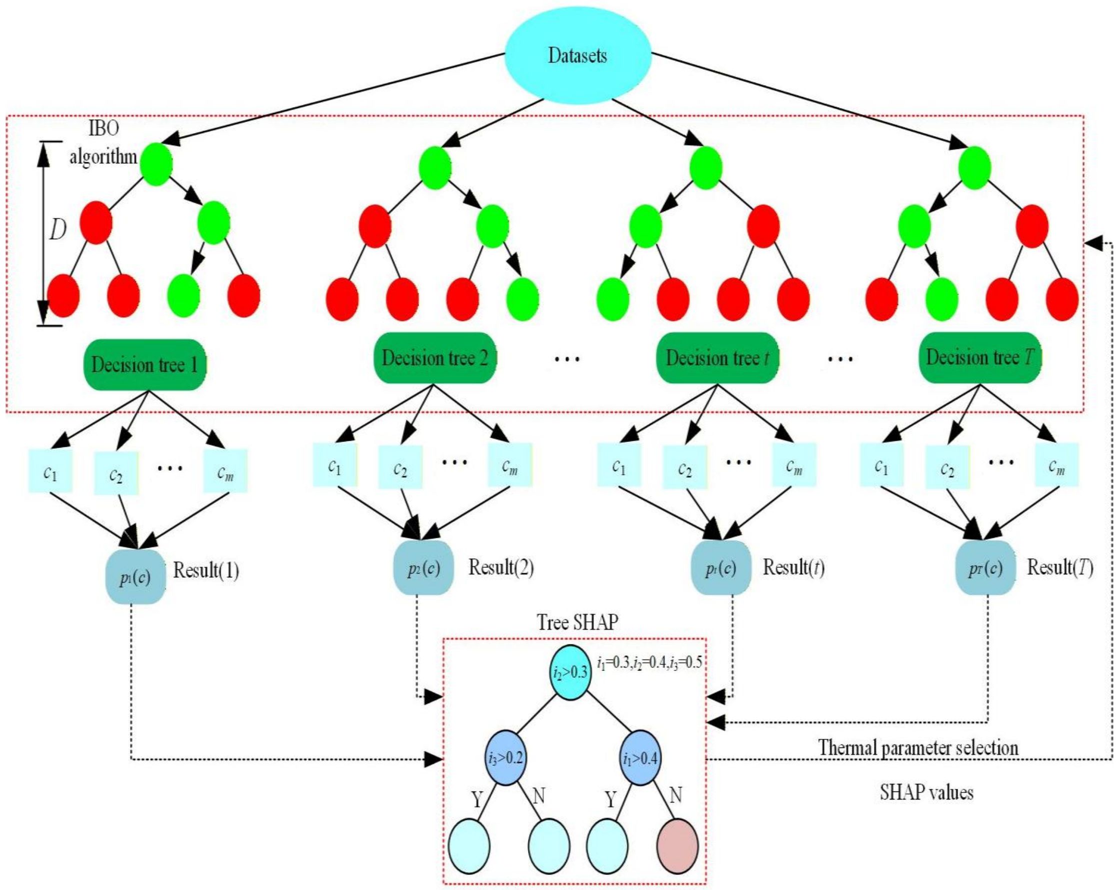


**Figure S1. Structure of the IBO-RF method.**

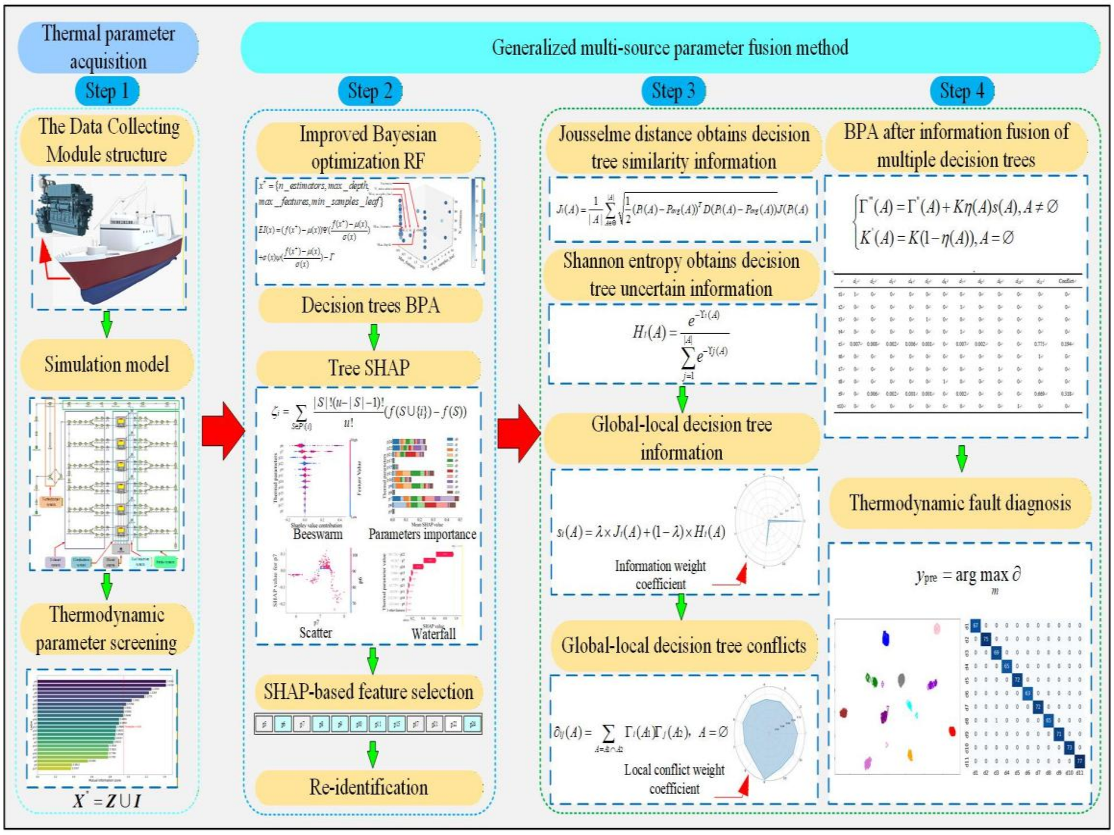


**Figure S2. The structure of IBO-RF-IDS.**

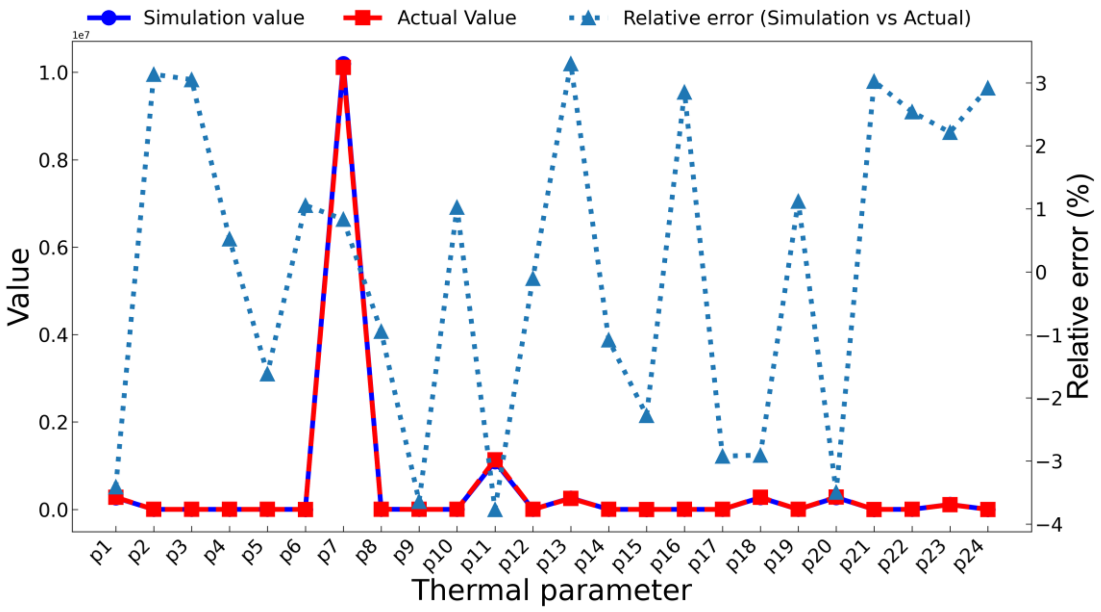


**Figure S3. Validation of simulation model validity.**

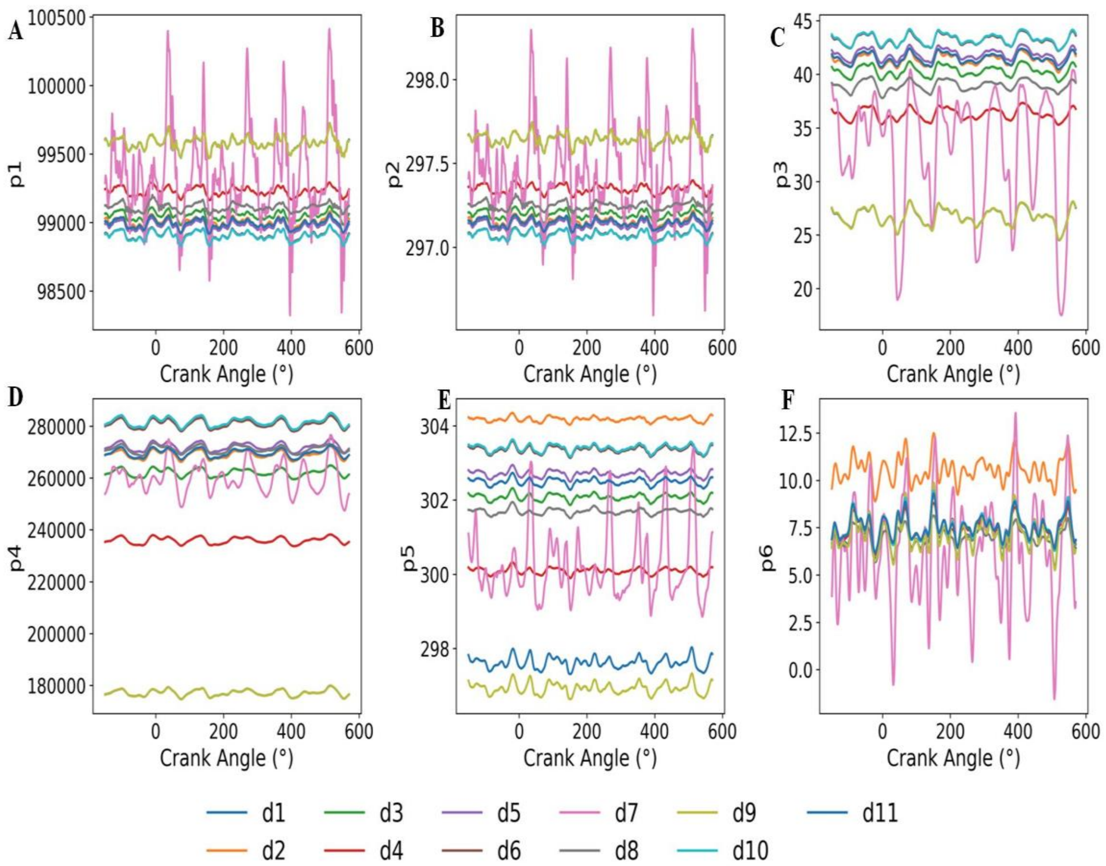


**Figure S4. Results of partial parametric fault simulation.**

(A) Pressure of Intake Gas after Turbocharger.

(B) Temperature of Intake Gas after Turbocharger.

(C) Velocity of Intake Gas after Turbocharger.

(D) Charge Air Temperature Before Intercooler.

(E) Charge Air Temperature After Intercooler.

(F) Intercooler Velocity.

**Table S1. Thermodynamic parameters of the simulation output**

| Parameters | Unit | Label | Parameters | Unit | Label |
|---|---|---|---|---|---|
| Pressure of Intake Gas after Turbocharger | Pa | p1 | Pressure of Exhaust Gas before Turbocharger | Pa | p13 |
| Temperature of Intake Gas after Turbocharger | K | p2 | Temperature of Exhaust Gas after Turbocharger | K | p14 |
| Velocity of Intake Gas after Turbocharger | $m \times s^{-1}$ | p3 | Pressure of Exhaust Gas after Turbocharger | Pa | p15 |
| Charge Air Temperature Before Intercooler | K | p4 | Velocity of Exhaust Gas after Turbocharger | $m \times s^{-1}$ | p16 |
| Charge Air Temperature After Intercooler | K | p5 | Temperature of Intake Manifold | K | p17 |
| Intercooler Velocity | $m \times s^{-1}$ | p6 | Pressure of Intake Manifold | Pa | p18 |
| Maximum Pressure During Combustion | Pa | p7 | Temperature of Exhaust Manifold | K | p19 |
| Maximum Temperature During Combustion | K | p8 | Pressure of Exhaust Manifold | Pa | p20 |
| Brake Power | kW | p9 | Velocity of Exhaust Manifold | $m \times s^{-1}$ | p21 |
| Brake Specific Fuel Consumption | $g \cdot KW^{-1} \cdot h^{-1}$ | p10 | Temperature of Exhaust | K | p22 |
| Brake Mean Effective Pressure | Pa | p11 | Pressure of Exhaust | Pa | p23 |
| Temperature of Exhaust Gas before Turbocharger | K | p12 | Flow Velocity of Exhaust | $m \times s^{-1}$ | p24 |

**Table S2. Details of fault parameters**

| Fault type | Label | Details | Fault type | Label | Details |
|---|---|---|---|---|---|
| Compressor blade fouling | d1 | Flow Multiplier:0.95 Efficiency Multiplier:0.95 | Early exhaust valve closure | d7 | Advance exhaust valve closing by 5–10 °CA |
| Cooler air-side blockage | d2 | Increase pressure loss coefficient by 50%-100% | Intake valve leakage | d8 | Equivalent leakage area 0.5–2 mm² |
| Injector timing advance | d3 | Advance injection start by 2–5 °CA | Turbine blade fouling | d9 | Flow Multiplier:0.95 Efficiency Multiplier:0.95 |
| Injector nozzle clogging | d4 | Reduce single-cycle injection by 10%-20% | Compression ratio reduction | d10 | Compression ratio reduced from 14.5:1 to 13.5:1 or 15.5:1 |
| Uneven single-cylinder fuel supply | d5 | Adjust injection of a single cylinder by ±15%, others unchanged | Normal condition | d11 | |
| Ignition delay | d6 | Delay combustion start by 3–6 °CA | | | |

**Table S3. Main parameters of diesel engine**

| Parameters | Value | Parameters | Value |
|---|---|---|---|
| Effective power /kw | 220 | Cylinder firing order | 1-5-3-6-2-4 |
| Speed / $r \cdot min^{-1}$ | 1000 | Number of cylinders | 6 |
| Bore /mm | 160 | Cylinder arrangement | Inline |
| Stroke /mm | 200 | Average effective pressure /bar | 16.4 |
| Compression ratio | 14.5 | Average piston speed / $m \cdot s^{-1}$ | 5.3 |
| Effective fuel consumption rate /( $g \cdot kw^{-1} \cdot h^{-1}$ ) | 191 | Connecting rod length /mm | 360 |

**Table S4. Model parameter settings**

| Methods | Parameter setting |
|---|---|
| Proposed method | n_estimators: 69, max_depth: 17, max_features: sqrt, $k$:0.7, $\lambda$ :0.5, acquisition function: EI, Kernel: Gaussian kernel, n_init:30 |
| DFCSSAES-WDS[a] | The optimized algorithm is Adam, learning rate is 0.0001, SAE-softmax5:560-280-140-70-7 |
| SDAE-BSD-DS[b] | Loss function: MSE and Binary Cross-Entropy, Layers, Activation function: ReLu, tanh, and sigmoid, Learning rate is 0.0001, Batch-size:128, $\lambda$ :0.5, $\|\cdot\|$ : Euclidean distance |
| MGCN-TMIF-DST[c] | k:5; Kernel function: Gaussian kernel; Learning rate is 0.0001; Loss function: CrossEntropyLoss; Activation: ReLU, Edges: Euclidean distance, Nodes: KNN, Normalization function: symmetry normalization, Hidden Units:16, Convolutional layers:3 |

[a]Gao et al. [16] .

[b]Wang et al. [17].

[c]Zhang et al. [15].

**Supplemental references**


15. Zhang, K., Li, H., Cao, S., Lv, S., Yang, C., and Xiang, W. (2023). Trusted multi-source information fusion for fault diagnosis of electromechanical system with modified graph convolution network. Adv. Eng. Inform. 57, 102088.

16. Gao, H., Zhang, X., Gao, X., Li, F., and Han, H. (2024). A hierarchical coarse-to-fine fault diagnosis method for industrial processes based on decision fusion of class-specific stacked autoencoders. IEEE T. Instrum. Meas. 73, 3528014.

17. Wang, Y., Zou, Y., Sun, W., Xiao, B., Hu, W., and Xiao, Z. (2025). Multi-source monitoring information fusion and state evaluation method of hydropower unit based on belief Sinkhorn distance. Measurement 253, 117581.
